# Profiling Germinal Center-like B Cell Responses to Conjugate Vaccines Using Synthetic Immune Organoids

**DOI:** 10.1021/acscentsci.2c01473

**Published:** 2023-04-12

**Authors:** Tyler D. Moeller, Shivem B. Shah, Kristine Lai, Natalia Lopez-Barbosa, Primit Desai, Weiyao Wang, Zhe Zhong, David Redmond, Ankur Singh, Matthew P. DeLisa

**Affiliations:** † Robert F. Smith School of Chemical and Biomolecular Engineering, Cornell University, Ithaca, New York 14853, United States; ‡ Nancy E. and Peter C. Meinig School of Biomedical Engineering, Cornell University, Ithaca, New York 14853, United States; § George W. Woodruff School of Mechanical Engineering, Georgia Institute of Technology, Atlanta, Georgia 30332, United States; ∥ Biochemistry, Molecular and Cell Biology, Cornell University, Ithaca, New York 14853, United States; ⊥ Institute for Computational Biomedicine, Weill Cornell Medicine, Cornell University, New York, New York 10021, United States; # Department of Physiology and Biophysics, Weill Cornell Medicine, Cornell University, New York, New York 10021, United States; ∇ Wallace H. Coulter Department of Biomedical Engineering, Georgia Institute of Technology, Atlanta, Georgia 30332, United States; ○ Cornell Institute of Biotechnology, Cornell University, Ithaca, New York 14853, United States

## Abstract

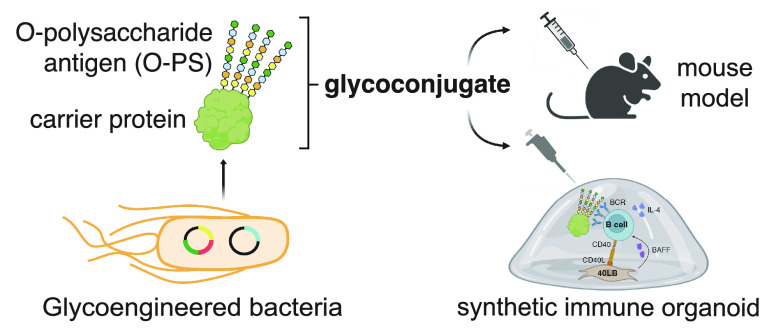

Glycoengineered bacteria have emerged as a cost-effective platform for rapid and controllable biosynthesis of designer conjugate vaccines. However, little is known about the engagement of such conjugates with naïve B cells to induce the formation of germinal centers (GC), a subanatomical microenvironment that converts naïve B cells into antibody-secreting plasma cells. Using a three-dimensional biomaterials-based B-cell follicular organoid system, we demonstrate that conjugates triggered robust expression of hallmark GC markers, B cell receptor clustering, intracellular signaling, and somatic hypermutation. These responses depended on the relative immunogenicity of the conjugate and correlated with the humoral response *in vivo.* The occurrence of these mechanisms was exploited for the discovery of high-affinity antibodies against components of the conjugate on a time scale that was significantly shorter than for typical animal immunization-based workflows. Collectively, these findings highlight the potential of synthetic organoids for rapidly predicting conjugate vaccine efficacy as well as expediting antigen-specific antibody discovery.

## Abstract 2

Here, we engineered mouse B cell follicle organoids to understand the impact of glycoconjugate vaccine candidates on B cell maturation and to identify high-affinity antigen-specific antibodies.

## Introduction

For decades, vaccines have been important pillars in preventative medicine, protecting against a wide array of disease-causing pathogens by inducing humoral and/or cellular immunity. Of the many possible candidate antigens for subunit vaccine development, carbohydrates are particularly appealing because of their ubiquitous presence on the surface of diverse pathogens such as bacteria, viruses, parasites, and even human cancers. In the case of pathogenic bacteria, high-molecular-weight polysaccharides in the form of capsular polysaccharides (CPS) and lipopolysaccharides (LPS) decorate the microbial exterior. Unfortunately, when free CPS or LPS antigens are administered as vaccines, they typically stimulate T-cell-independent humoral immune responses, but such responses are relatively weak.^1^ These responses are characterized by a lack of IgM-to-IgG class switching in B cells, failure to induce a secondary antibody response after recall immunization, and no sustained T-cell memory.^2^ T-cell-independent polysaccharide antigens can be readily converted into more potent immunogens by covalent conjugation to a CD4^+^ T-cell-dependent antigen such as an immunostimulatory protein carrier.^3,4^ Indeed, conjugate vaccines composed of CPS or LPS-based antigens chemically bound to the *Clostridium tetani* tetanus toxin (TT) or the *Corynebacterium diphtheriae* diphtheria toxin (DT) induce polysaccharide-specific IgM-to-IgG switching, memory B cell development, and long-lived T-cell memory.^4,5^ Such conjugates have proven to be a highly efficacious and safe strategy for protecting against virulent pathogens, including *Haemophilus influenzae*, *Neisseria meningitidis*, and *Streptococcus pneumonia*. This effective glycoconjugate vaccine format is used in currently approved pneumococcal, meningococcal, and *Haemophilus influenzae* type B vaccines, and several recent reviews discuss the mechanism of such glycoconjugate vaccines in detail.^4,6^

The robustness of B cell activation induced by a polysaccharide-carrier conjugate depends on a variety of factors including carrier immunogenicity, location of glycan attachment, and glycan composition and size, all of which are known to modulate the immune response as characterized by serum titer strength and elicitation of protective antibodies. Such antibodies are produced when B cells bind the antigen and initiate the formation of tightly regulated transient germinal center (GC) structures in cooperation with other immune and stromal cells, followed by immunoglobulin isotype class-switching.^7,8^ Within the GC, B cell programming includes somatic hypermutation (SHM) and multiple rounds of selection to rapidly increase binding affinity and promote differentiation into antibody-secreting cells and memory cells. Eliciting GCs and their ensuing high-affinity antibodies, as well as understanding GC immunobiology and the interactions of conjugate vaccine components with naïve B cells as they undergo maturation, is central to the design of better polysaccharide antigens and carriers. However, due to the complexity of GC formation with its myriad of cellular, chemical, and physical signals, animal models are the standard means of eliciting antibody responses to empirically evaluate vaccine immunogenicity and efficacy. While insightful, animal immunization is cost-prohibitive, time-consuming, and has a relatively low throughput. Moreover, these issues are at odds with newer preparation methods based on glycoengineered bacteria or their cell-free extracts that enable facile biosynthesis of conjugate vaccine candidates^9,10^ with greater control over critical design parameters and the potential for generating large libraries.^11^ Consequently, there is a need for new tools that allow rapid screening of potentially large libraries of different glycoconjugate configurations to identify immunologically superior designs that can be down-selected for resource-intensive animal studies.

Over the past decade, the rise of biomaterials in tissue engineering has afforded intriguing new opportunities to explore B cell activation and maturation. Moving beyond traditional stimulation and differentiation of B cells *in vitro*, immune tissues create a 3D cell culture architecture that more closely mimics native conditions by encapsulating B cells, costimulatory signals, and cytokines in a hydrogel matrix.^12,13^ Recent work using a bottom-up organoid tissue approach was able to faithfully recapitulate key phenotypes of GC B cells, including GC-relevant RNA and gene expression profiles that closely resembled *in vivo* GC B cells.^14^ As occurs during SHM *in vivo*, significant increases in expression of *aicda*, which encodes activation-induced cytidine deaminase (AID), and immunoglobulin variable loci mutation rate were observed in B cells after organoid incubation. Building upon this finding, Purwada et al. characterized the response of immune organoids after addition of the small molecule hapten 4-hydroxy-3-nitrophenylacetyl (NP) conjugated to ovalbumin.^15^ NP-specific B cells were identified from organoids prepared with B cells derived from wild-type mice. Due to the GC-like phenomena of engineered immune organoids, they are a compelling platform to investigate intricacies of the humoral immune response to an infection or vaccination in a timely and scalable manner.^16^ Immune organoid culture systems not only provide an accurate immune tissue microenvironment to model GC-like behavior, but are also high-throughput. A single mouse spleen is sufficient to prepare >800 organoids, which can be arrayed in 96-well plates for high-throughput experimentation and analyzed after 4 days of incubation as compared to the weeks or months normally needed for animal immunization work.

Here, we engineered and characterized murine B cell follicle organoids to systematically understand the impact of glycoconjugate vaccine candidates on B cell maturation and signaling. Since polysaccharide antigens and carriers exhibit differing degrees of immunogenicity *in vivo* as measured by the magnitude and protective efficacy of the elicited antibodies, we hypothesized that organoids potentiated by different antigens would also exhibit differences in measurable outputs. To test this hypothesis, we developed an experimental framework in which glycoconjugate vaccine candidates derived from glycoengineered *Escherichia coli* cells were immunologically evaluated *in vivo* using mice and *ex vivo* using our recently reported hydrogel-based immune organoids.^14,15^ Importantly, the *ex vivo* GC responses were observed to (i) depend on the relative immunogenicity of the carrier protein, (ii) increase further by the addition of an O-polysaccharide (O-PS) antigen, and (iii) correlate with the humoral responses *in vivo.* In a more application-oriented goal, the occurrence of these mechanisms in immune organoids was exploited for identifying antigen-specific antibodies. Specifically, we screened antibody repertoires of GC-like B cells derived from antigen-exposed organoids using yeast surface display (YSD) and discovered high-affinity antibodies against both carrier and O-PS antigens. By sidestepping animal immunization, this immune organoid-based approach enabled antibody discovery on a time scale that was significantly shorter than for conventional immunization-based workflows.

## Results

### Biosynthesis of Designer Glycoconjugate Vaccine Candidates

**Figure 1:**
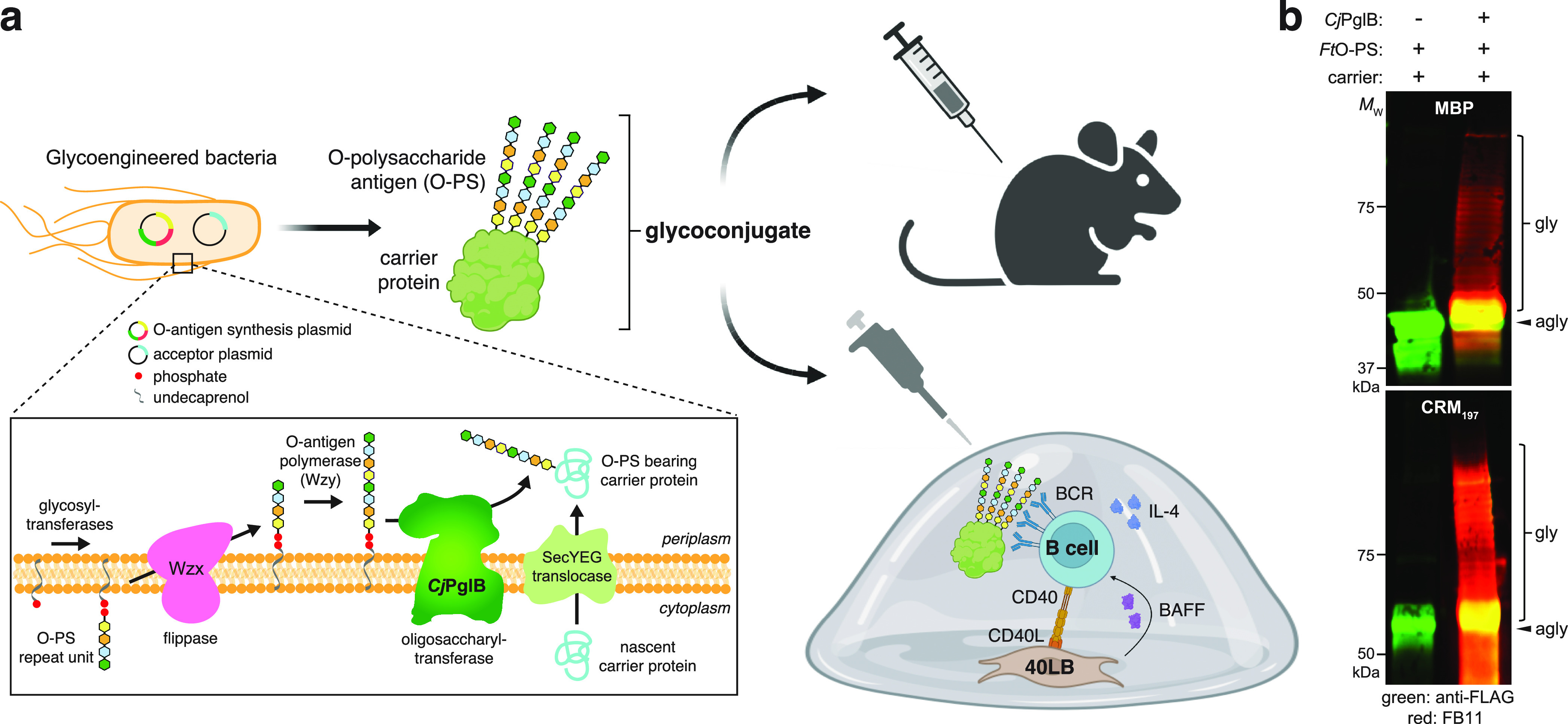
Experimental framework for profiling GC-like B cell responses to glycoconjugate vaccine candidates. (a) Schematic of strategies for preparing designer glycoconjugate vaccine candidates (left) and evaluating their immunogenicity in mice and in synthetic immune organoids (right). Glycoengineered bacteria enable the assembly of a single O-PS repeat unit (a tetrasaccharide structure in the case of *F. tularensis* Schu S4) on a undecaprenol lipid carrier in the cytoplasmic membrane, which is subsequently flipped into the periplasm and polymerized to form variable-length O-PS antigens by the endogenous Wzx flippase and Wzy O-antigen polymerase Wzy, respectively. In the presence of *C. jejuni* PglB (*Cj*PglB), the lipid-linked O-PS is site-specifically transferred to asparagine residues in recombinant carrier proteins bearing a DQNAT motif. Schematic created with BioRender.com. (b) Immunoblot analysis of purified carrier proteins derived from *E. coli* CLM24 cells carrying a plasmid encoding either MBP^4xDQNAT^ or CRM_197_^4xDQNAT^ along with plasmid pGAB2 encoding the *Ft*O-PS biosynthetic pathway and with (+) or without (−) plasmid pMAF10 encoding *Cj*PglB as indicated. Blots were probed with anti-FLAG antibody to detect acceptor proteins (green signal) and FB11 antibody to detect *Ft*O-PS antigens (red signal). Images depict an overlay of anti-FLAG and FB11 blots. Arrows denote aglycosylated (agly) and multiply glycosylated (gly) forms of MBP^4xDQNAT^ and CRM_197_^4xDQNAT^. Molecular weight (*M*_W_) markers are indicated on the left. Results are representative of three biological replicates.

Candidate glycoconjugates were generated using bacterial glycoprotein expression technology,^9^ which enabled covalent conjugation of pathogen-specific polysaccharides to specific sites in a carrier protein. Specifically, we leveraged engineered *E. coli* cells that were capable of assembling heterologous O-PS antigens on the cytoplasmic membrane lipid undecaprenol and subsequently transferring lipid-linked O-PS onto specific asparagine residues in recombinantly expressed carrier proteins via the activity of the oligosaccharyltransferase (OST) enzyme PglB from *Campylobacter jejuni* (Figure 1a). For the pathogen-specific polysaccharide component, we focused on the O-PS antigen of *Francisella tularensis* (type A) strain Schu S4 (*Ft*O-PS), a highly virulent pathogen that causes tularemia and for which there is no currently approved vaccine.^17^ The choice of *Ft*O-PS was motivated by the fact that this glycan has previously been produced as a protein-linked conjugate using engineered *E. coli* or its cell-free extracts.^18−20^ When evaluated *in vivo*, the resulting conjugates elicited strong *Ft*O-PS-specific IgG antibody titers that were protective against lethal doses of *F. tularensis*.^18−20^

For the carrier protein component, we investigated two different designs. The first involved the most widely used and highly effective carrier protein cross-reactive material 197 (CRM_197_), a mutant version of the diphtheria toxin carrying a single amino acid substitution (G52Ε) that renders the protein nontoxic.^21^ CRM_197_ is used in several licensed conjugate vaccines including HibTITER, Prevnar, and Menveo. The second involved *E. coli* maltose-binding protein (MBP), which has demonstrated compatibility with bacterial protein glycosylation technologies.^18,22,23^ While not a prototypic carrier, MBP might enhance immune responses to vaccine fusion proteins or conjugated polysaccharides on account of its ability to induce dendritic cell activation and production of proinflammatory cytokines.^24^ Indeed, when linked to O-PS, MBP was found to elicit polysaccharide-specific humoral and cellular immune responses in mice^18,23^ including against *Ft*O-PS, with the latter protecting against pathogen challenge.^18^ However, little is known about the interactions of these carrier proteins and conjugated antigens with B cells undergoing GC differentiation.

To enable conjugation with *Ft*O-PS, both CRM_197_ and MBP carriers were engineered with a C-terminal tag containing four tandem repeats of an optimized bacterial glycosylation acceptor motif, DQNAT, that is preferentially glycosylated by *C. jejuni* PglB.^25^ Additional C-terminal polyhistidine and FLAG (DYKDDDDK) epitope tags were also introduced to enable purification and immunoblot detection, respectively. Following expression in *E. coli* strain CLM24 that carried plasmids encoding the *Ft*O-PS biosynthetic pathway and *C. jejuni* PglB, both carrier proteins were purified and found to be efficiently glycosylated with heterologous *Ft*-O-PS as evidenced by the appearance of a ladder-like banding pattern in immunoblots probed with either anti-His antibody or an antibody, anti-*Ft*LPS, against pathogen-derived *F. tularensis* lipopolysaccharide (Figure 1b). This ladder was characteristic of *Ft*O-PS attachment and reflected O-PS chain length variability through the action of the O-antigen polymerase,Wzy, responsible for adding O-PS repeat units to lipid-A core.^18,19,26^ In contrast, when *C. jejuni* PglB was absent from cells, no *Ft*O-PS glycan-specific signal from immunoblots was detected (Figure 1b), confirming the lack of protein glycosylation.

### Glycoconjugates Differentially Elicit Antigen-Specific Antibodies in Vivo

**Figure 2:**
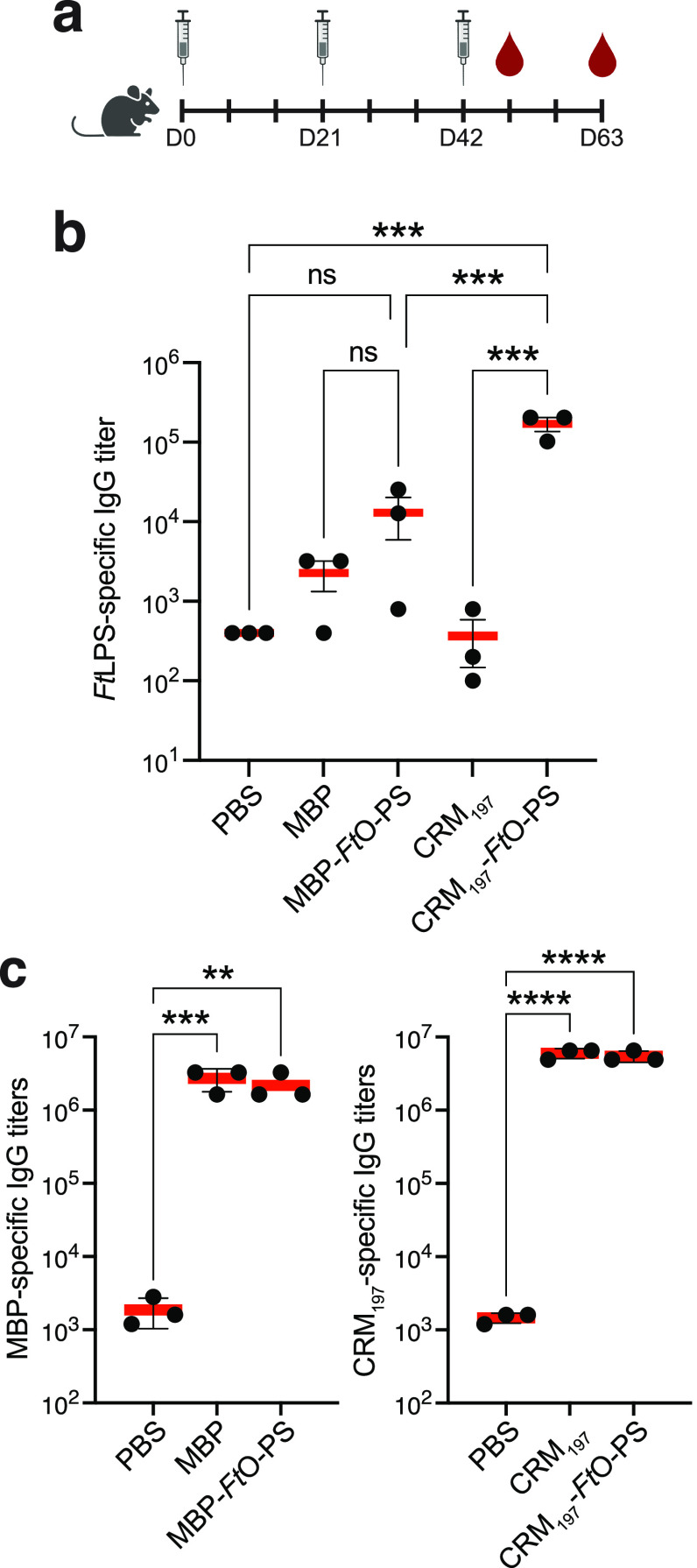
Glycoconjugates differentially boost antigen-specific IgG titers *in vivo*. (a) Schematic of the prime-boost immunization schedule. Mice received an initial injection on day 0 (D0) and identically formulated booster injections on days 21 and 42. Blood was drawn on days 49 and 63. (b) *Ft*LPS-specific IgG titers in day 63 serum of individual mice (black dots) and mean titers of each group (red lines) as determined by ELISA with *Ft*LPS as immobilized antigen. Groups of three BALB/c mice were immunized s.c. with 100 μL of PBS alone or PBS containing 10 μg of glycoconjugate (MBP-*Ft*O-PS or CRM_197_-*Ft*O-PS) adjuvanted with IFA or 10 μg of aglycosylated carrier protein (MBP or CRM_197_) adjuvanted with IFA. Mice were boosted on days 21 and 42 with the same doses. For *Ft*LPS-specific IgG titers at day 49, see Supplementary Figure S1. (c) Carrier protein-specific serum IgG titers in day 63 serum of individual mice (black dots) determined as in (a) but with MBP (left panel) and CRM_197_ (right panel) as immobilized antigens. For *Ft*LPS-specific IgG titers at day 49, see Supplementary Figure S1. Significant differences were determined via one-way ANOVA with Tukey’s posthoc test (*p < 0.05, **p < 0.01, ***p < 0.001, and ****p < 0.0001; ns, not significant).

To benchmark the immunogenicity of the two different glycoconjugate formulations, we performed conventional animal immunizations. Specifically, BALB/c mice were immunized subcutaneously with 10 μg (on a total protein basis) of purified conjugates or aglycosylated carrier protein controls lacking *Ft*O-PS antigens, all of which were adjuvanted with Incomplete Freund’s Adjuvant (IFA). Following the initial injections, identically prepared booster doses were given subcutaneously at 21 and 42 days, and blood was drawn at day 49 and 63 (Figure 2a). Serum IgG titers specific for *Ft*LPS, which includes the O-PS component, were significantly increased in mice receiving the CRM_197_ conjugate after the first booster with relatively little change in titers following the second booster (Figure 2b and Supplementary Figure S1), consistent with titers measured previously for *Ft*O-PS-based glycoconjugates.^18^ The *Ft*LPS-specific IgG titers elicited by the MBP conjugate were significantly lower compared to the CRM_197_ conjugate, which may have been due to the more extensive polysaccharide decoration observed on CRM_197_ relative to its MBP counterpart (Figure 1b). Another possible explanation that we considered was that the CRM_197_ carrier might be more immunogenic than the MBP carrier. Nevertheless, strong serum IgG titers specific to the MBP and CRM_197_ carrier proteins were elicited by the respective glycoconjugates; with each boosting, carrier protein-specific IgG titers were approximately 3 logs above the background titers measured for mice receiving PBS (Figure 2c and Supplementary Figure S1). Importantly, the distinct humoral responses observed for the MBP and CRM_197_ conjugates suggested that these would be a useful set of reagents for interrogating the ability of synthetic immune organoids to discriminate conjugate vaccine immunogenicity.

### Glycoconjugate-Exposed Organoids Exhibit Enhanced GC-like Responses

**Figure 3:**
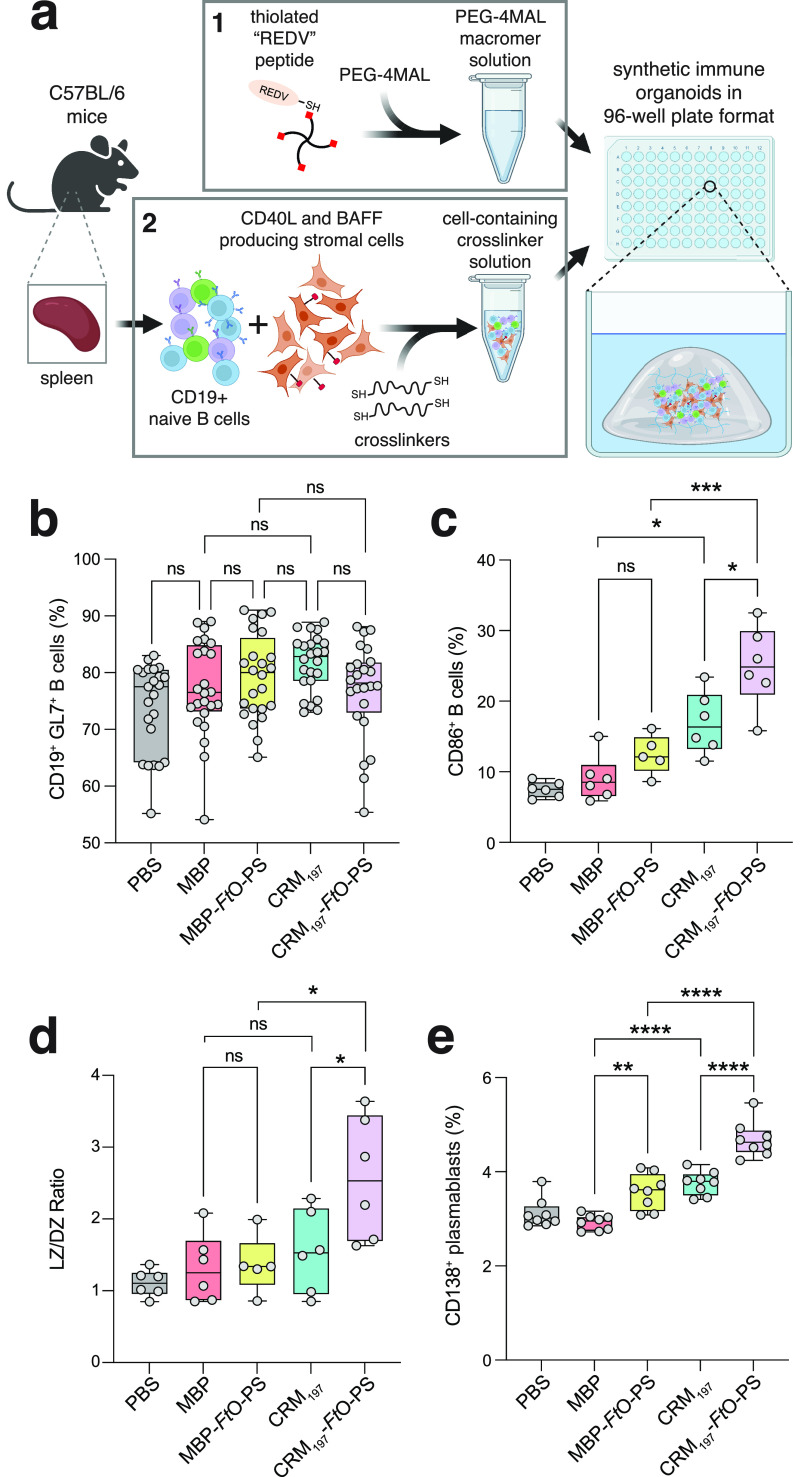
Glycoconjugates shape the GC-like B cell subpopulations in synthetic organoids. (a) Schematic of *ex vivo* lymphoid immune organoids. B cells are isolated from spleens of C57BL/6 mice and encapsulated with CD40L-presenting fibroblasts in protease-degradable PEG-4MAL hydrogels functionalized with bioadhesive REDV peptide. Schematic created with BioRender.com. Quantitative flow cytometric analysis of (b) CD19^+^ GL7^+^ GC-like B cells, (c) CD86^+^ GC-like B cells, (d) ratio of CXCR4^lo^CD86^hi^ (LZ) and CXCR4^hi^CD86^lo^ (DZ) GC-like B cells, and (e) CD138^+^ plasmablasts. Organoids were exposed to either PBS or PBS containing 10 μg of glycoconjugate (MBP-*Ft*O-PS or CRM_197_-*Ft*O-PS) or 10 μg of aglycosylated carrier protein (MBP or CRM_197_). Data represent the mean ± standard error of the mean (SEM). See Supplementary Figures S2 and S3 for all flow cytometric gating strategies used to identify GC-like B cell subpopulations. Significant differences were determined via one-way ANOVA with Tukey’s posthoc test (*p < 0.05, **p < 0.01, ***p < 0.001, and ****p < 0.0001; ns, not significant; *n* = 24 in panel b; *n* = 6 in panels c and d; *n* = 8 in panel e).

We hypothesized that the MBP and CRM_197_ glycoconjugates elicited these different humoral immune responses *in vivo* by distinctly modulating the phenotypic and transcriptional regulatory characteristics of primary GC B cells. To test this hypothesis, we leveraged our recently developed designer 3D murine B cell immune organoid culture system that induces GC reactions *ex vivo* in a controlled manner.^13−15,27−30^ Here, primary B cells from the spleens of nonimmunized wild-type mice were isolated and combined with stromal 40LB cells,^30,31^ which are murine 3T3 fibroblasts genetically engineered to express T cell signal CD40L and follicular dendritic cell signal B cell activation factor (BAFF) (Figure 3a), prior to coencapsulation in maleimide-functionalized polyethylene glycol (PEG-4MAL) hydrogels. This was achieved by first functionalizing PEG-4MAL hydrogels with thiolated integrin-binding peptide REDV, which mimics the extracellular matrix fibronectin domain and vascular cell adhesion molecule (VCAM-1) on follicular dendritic cell networks^28^ found within B cell follicles where GCs form. After functionalization, PEG-4MAL droplets were plated on a 96-well plate, mixed with an equal volume of cell-containing cross-linker solution, and cured at 37 °C to form hydrogels. Organoids were designed for a 96-well format to facilitate high-throughput and scalable studies by simplifying cell culture, addition of antigen, imaging, and processing for downstream analysis. This approach was previously shown to induce a robust early GC-like phenotype (CD19^+^ GL7^+^) in a ligand concentration-dependent manner^32^ as well as several hallmark epigenetic and transcriptional regulators of the GC process.^28^ Recombinant murine IL4, alongside the immunogen of interest, was added to the media where the hydrogel capsules were embedded. After 4 days, the hydrogels were enzymatically digested, and all cells were analyzed using flow cytometry. B cells displaying a GC-like (CD19^+^ GL7^+^) phenotype were isolated from the organoid hydrogel. Approximately similar amounts of GC-like B cells (∼80% of the total B cell population) were recovered from organoids regardless of the immunogen treatment (Figure 3b and Supplementary Figure S2 and S3), consistent with previous work.^15,32^ Because CD40L is provided by 40LB cells in PBS control groups, we expected high levels of GC-like B cells; however, our previous work demonstrates that these are not antigen-specific cells.^15^

We next sought to characterize the abundance of B cell populations known to be relevant for achieving robust humoral immunity. B cells within the GC are compartmentalized into two anatomically distinct compartments: the dark zone (DZ), representing the site of intense B cell proliferation and SHM; and the light zone (LZ), where B cells bind antigen and undergo selection aided by the presence of follicular helper T cells and antigen-presenting follicular dendritic cells.^7,8^ B cells that do not bind antigens and do not receive survival signals from these auxiliary cells undergo apoptosis. When B cells are primed with antigens and CD40L *in vivo*, GC B cells segregate into centroblasts (CXCR4^hi^ CD86^lo^) in the DZ and centrocytes (CXCR4^lo^ CD86^hi^) in the LZ, prior to selection and exit of the GC response. Indeed, flow cytometric analysis on GC-like B cells indicated significant upregulation of CD86, which is a marker of B cell activation, in CRM_197_-*Ft*O-PS conjugate as compared to MBP-*Ft*O-PS conjugate or either of the aglycosylated carrier proteins (Figure 3c and Supplementary Figure S2). Interestingly, the CRM_197_ carrier protein induced a significantly higher fraction of CD86^+^ B cells than MBP alone, demonstrating a distinct carrier effect on B cell maturation. Lastly, MBP or CRM_197_ glycoconjugates differentially modulated the centrocyte phenotype, where the CRM_197_-*Ft*O-PS conjugate induced a significantly higher percentage of LZ-like (CXCR4^lo^ CD86^hi^) B cells than MBP-*Ft*O-PS conjugate or aglycosylated CRM_197_ carrier protein (Figure 3d). In contrast, we observed no significant differences between glycosylated and aglycosylated MBP or between the two aglycosylated carrier proteins.

The downstream effect of B cell maturation is the generation of CD138^+^ plasmablasts. Similar to the centrocyte phenotype, we observed that the CRM_197_-*Ft*O-PS conjugate induced a significantly higher percentage of CD138^+^ plasmablasts compared to the MBP-*Ft*O-PS conjugate or aglycosylated carrier proteins (Figure 3e and Supplementary Figure S2). However, unlike centrocytes, the MBP-*Ft*O-PS conjugate induced a significantly higher percentage of CD138^+^ cells than aglycosylated MBP. The carrier effect on B cell terminal differentiation was again observed, with aglycosylated CRM_197_ inducing a significantly higher fraction of CD138^+^ cells than aglycosylated MBP. Taken together, these results indicate that while most organoid B cells were GC-like B cells for all groups, those from organoids treated with the CRM_197_ glycoconjugate exhibited the highest amounts of proteins representative of both GC-associated activation as well as differentiated LZ-like B cells, indicating a shift toward CD138^+^ plasmablast populations. These results were significant when compared to organoids treated with CRM_197_ carrier protein alone or with the less immunogenic MBP glycoconjugate.

### Glycoconjugates Shape BCR Clustering and Signaling in GC-like B Cells

**Figure 4:**
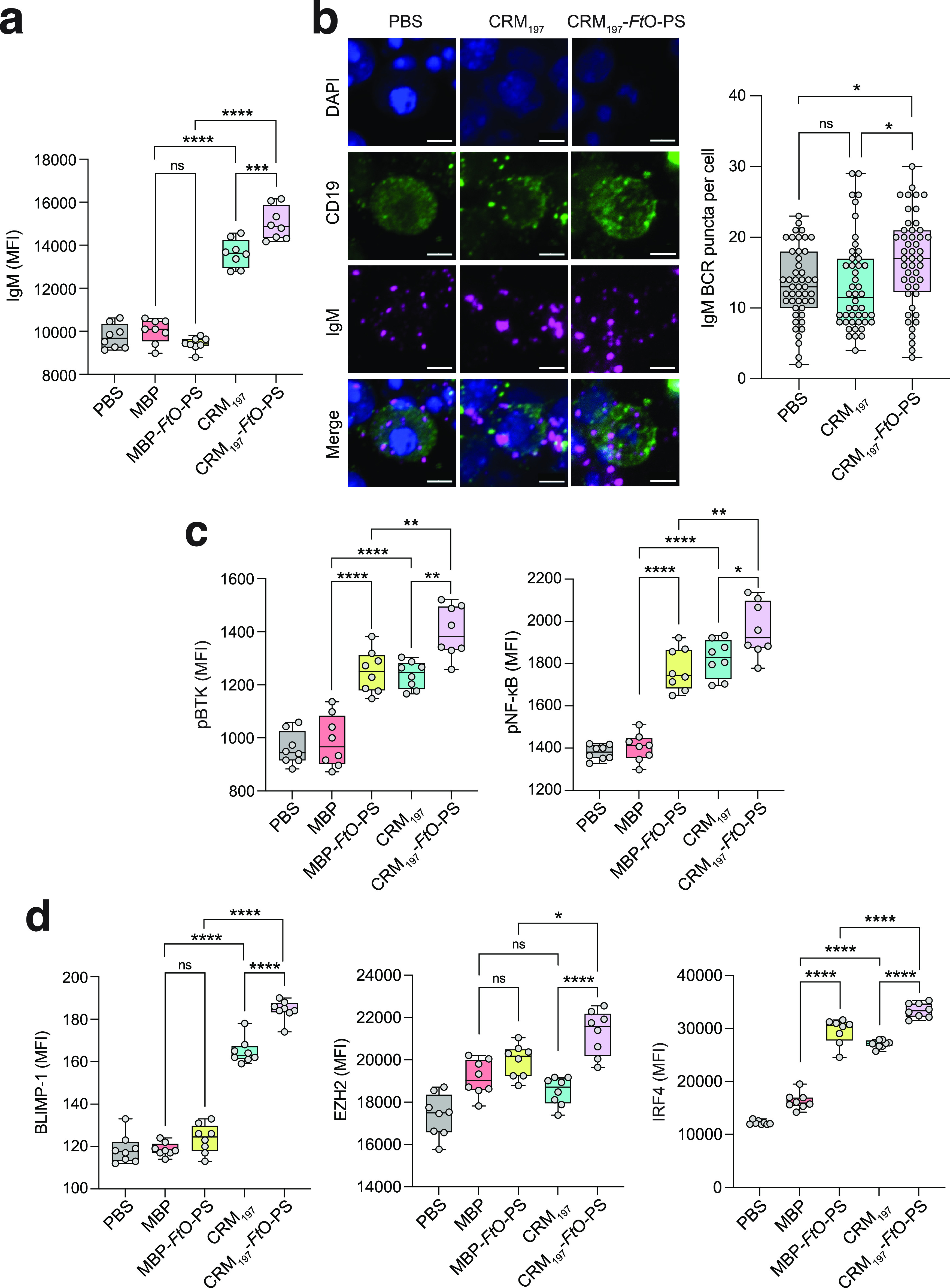
Glycoconjugates shape BCR clustering and signaling of GC-like B cells in synthetic organoids. (a) Quantitative flow cytometric analysis of CD19^+^ GL7^+^ GC-like B cells for expression of IgM after 4 days of *in vitro* culture. (b) Single-cell images of CD19^+^ B cells with IgM BCR puncta. Staining of nuclei was performed with 4′,6-diamidino-2-phenylindole (DAPI). Scale bars are 5 μm. Shown at right is the number of IgM BCR puncta per cell quantified from single cell images. (c, d) CD19^+^ GL7^+^ GC-like B cells for expression of (c) phosphorylated BTK (pBTK) and phosphorylated NF-κB (pNF-κB) and (d) BLIMP-1, EZH2, and IRF4 after 4 days of *ex vivo* culture. Synthetic immune organoids were exposed to either PBS or PBS containing the following: 10 μg of glycoconjugate (MBP-*Ft*O-PS or CRM_197_-*Ft*O-PS) or 10 μg of aglycosylated carrier protein (MBP or CRM_197_). Data represent the mean ± standard error of the mean (SEM). Values are reported as geometric mean fluorescence intensity (MFI). Significant differences were determined via one-way ANOVA with Tukey’s posthoc test (*p < 0.05, **p < 0.01, ***p < 0.001, and ****p < 0.0001; ns, not significant; *n* = 8). See Supplementary Figure S3 for flow cytometric gating strategies used to identify GC-like B cell responses.

Another key facet of the humoral immune response is antigen binding to BCRs, which initiate intracellular signaling that promotes a GC response.^33,34^ Upon binding of antigen, B cell activation is regulated by BCR signaling and the nanoscale organization of the BCR on the cell surface. These nanoscale organizations lead to the formation of individual puncta on the surface of B cells. We hypothesized that organoids treated with the CRM_197_ glycoconjugate would exhibit higher BCR clusters than other comparative groups. To test this hypothesis, we quantified IgM expression on GC-like B cells and imaged BCR puncta on B-cell surfaces. The CRM_197_-*Ft*O-PS conjugate significantly upregulated IgM expression on activated B cells as compared to MBP-*Ft*O-PS conjugate or either of the aglycosylated carrier proteins as determined using mean fluorescence intensity (Figure 4a). In contrast, these were not a significant increase in IgM expression for the MBP-*Ft*O-PS conjugate or MBP carrier relative to the PBS group. The IgM expression was further impacted by the carrier effect, whereby aglycosylated CRM_197_ carrier induced significantly higher IgM fluorescence than aglycosylated MBP. Spatially, the CRM_197_-*Ft*O-PS conjugate induced the formation of a significantly higher number of BCR clusters per single B cell than the aglycosylated CRM_197_ carrier (Figure 4b), indicating that the glycoconjugate functions by enhanced BCR expression and clustering.

We hypothesized that the different immunostimulatory effects of the two glycoconjugates on B cells undergoing maturation in *ex vivo* immune organoids would involve distinct activation of signaling in B cells. Specifically, using flow cytometry, we measured the levels of activated Bruton’s tyrosine kinase (BTK), an intermediary kinase, and nuclear factor-κB (NF-κB), a downstream transcription factor responsible for important B cell maturation processes including class-switching, in the GC-like B cell population. GC-like B cells derived from organoids exposed to CRM_197_, with and without conjugation to *Ft*O-PS, exhibited increased staining of phosphorylated BTK (pBTK) and phosphorylated NF-κB (pNF-κB) relative to organoids exposed to their respective MBP counterparts (Figure 4c and Supplementary Figure S3). Importantly, the observation that glycosylated CRM_197_ stimulated greater activation of BTK and NF-κB was consistent with its stronger immunogenicity *in vivo*. To our surprise, both the CRM_197_ and MBP glycoconjugates stimulated a statistically significant increase in pBTK and pNF-κB levels relative to their cognate aglycosylated carrier proteins, suggesting a role for the conjugated *Ft*O-PS antigen itself in further activation of BTK and NF-κB.

Next, we examined the expression levels of factors associated with the GC response, including BLIMP-1, EZH2, and IRF4. Both EZH2 and IRF4 are upstream of multiple GC programming factors and have been shown to be required for GC formation.^14,35,36^ During the late stages of the GC response, IRF4 levels are again elevated and BLIMP-1 is also expressed, with both working together and required for GC B cell differentiation into plasma cells.^36^ For all three of these GC factors, glycosylated CRM_197_ induced significantly greater expression relative to all other treatment groups including both the aglycosylated CRM_197_ carrier and the glycosylated MBP conjugate (Figure 4d and Supplementary Figure S3). In contrast, treatment with glycosylated and aglycosylated MBP did not lead to any statistically meaningful differences in BLIMP-1 and EZH2 expression levels. Only in the case of IRF4 did glycosylated MBP trigger higher expression relative to aglycosylated MBP. Interestingly, both the glycosylated and aglycosylated CRM_197_ immunogens stimulated greater expression of BLIMP-1 and IRF4 relative to their respective MBP counterparts. Taken together, these results provide further support that the *Ft*O-PS component of the conjugate promotes activation of GC-like responses, which were most pronounced in the context of the CRM_197_ carrier protein.

### Glycoconjugate-Exposed Organoids Exhibit Increased BCR Engagement by Immunogens

**Figure 5:**
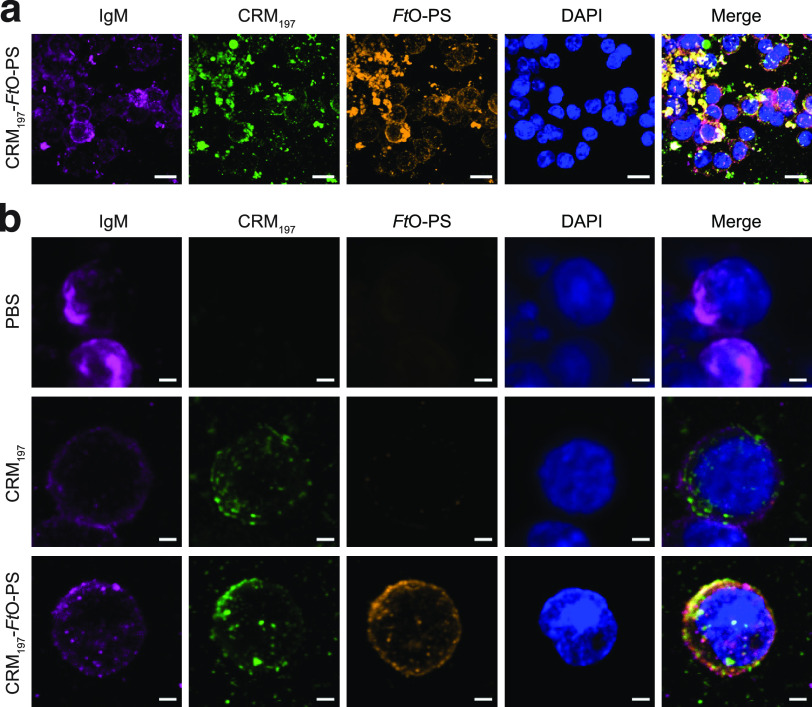
BCR clustering on immunogen-exposed GC-like B cells. Representative images of B cells in immune organoids exposed to PBS, PBS containing CRM_197_, or PBS containing CRM_197_-*Ft*O-PS after 4 days of culture. (a) Cell clusters in organoids. Scale bars are 10 μm. (b) Single cell images of B cells showing the presence or absence of CRM_197_ and *Ft*O-PS. Staining of nuclei was performed with 4′,6-diamidino-2-phenylindole (DAPI). Scale bars are 2 μm. Merge represents IgM, CRM_197_, *Ft*O-PS, and DAPI.

We hypothesized that differences observed above in GC-like B cell outputs, in particular the levels of pBTK and pNF-κB that are known to be activated via BCR signal transduction, could reflect varying degrees of BCR engagement by the different immunogens. To test this hypothesis, we attempted to visualize CRM_197_-based immunogen binding to BCRs on the surface of organoid B cells. These immunogens were chosen because of their superior GC-like responses compared to the respective MBP immunogens. Following 4 days of *ex vivo* culture, GC-like organoid B cells were stained with antibodies specific for BCR, CRM_197_, and *F. tularensis* LPS and subsequently imaged by confocal microscopy. Our imaging analysis revealed that all three signals were present on the surface of CRM_197_ glycoconjugate-exposed organoid B cells (Figure 5a). Higher magnification at the single cell level revealed a clear overlap of the BCR, CRM_197_, and *Ft*O-PS signals indicative of BCR and glycoconjugate colocalization (Figure 5b). Interestingly, B cells associated with PBS-treated organoids had a diffuse BCR signal throughout the membrane, while B cells from organoids exposed to CRM_197_-based immunogens had distinct BCR puncta, consistent with the well-known phenomenon of antigen-induced BCR clustering that potentiates intracellular signaling through phosphorylation of immunoreceptor tyrosine-based activation motifs present in the cytoplasmic tail of BCR-associated proteins.^33^

### Antigen-Exposed Organoids Exhibit Diversification of Immunoglobulin Expression

**Figure 6:**
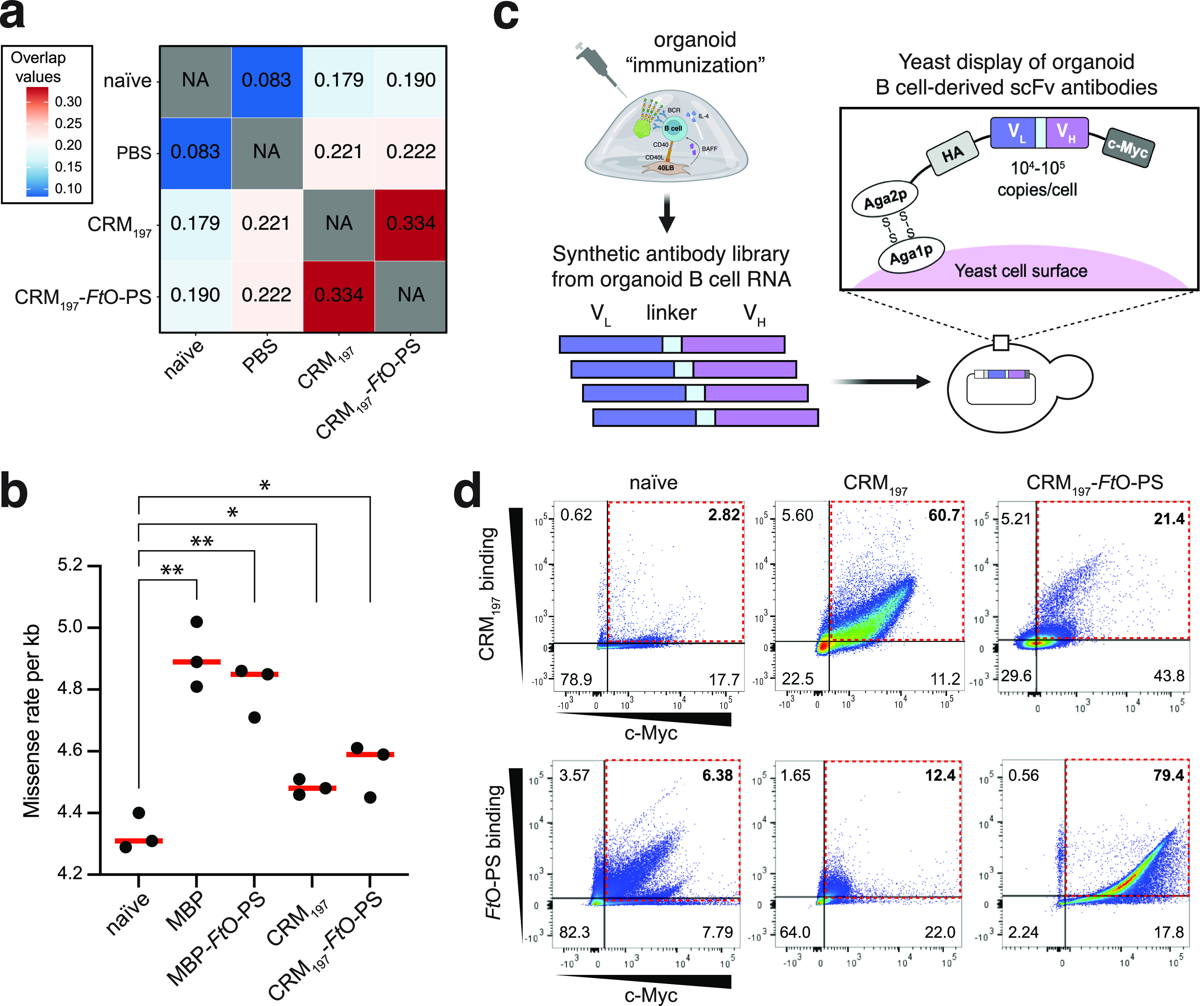
GC-like organoid B cell immunoglobulin repertoire analysis. (a) Sequence convergence of V_H_ repertoires as measured by Morisita’s overlap index. Libraries of V_H_ genes were prepared from RNA of naïve B cells from day 0 prior to organoid culture (naïve) and after 4 days of organoid culture treated with PBS, CRM_197_ carrier protein, or CRM_197_-*Ft*O-PS glycoconjugate. (b) SHM analysis performed by sequencing the Sμ immunoglobulin variable locus from organoid GC-like B cells. Data are the mean of three biological replicates. Statistical significance was determined by Welch’s two-sided *t*-test (*p < 0.05, **p < 0.01). (c) Schematic of YSD strategy for antibody repertoire analysis of GC-like organoid B cells. Libraries of V_H_ and V_L_ genes from organoid B cells were assembled as single-chain Fv (scFv) antibodies in which V_L_ genes were randomly paired to V_H_ genes via a flexible GlySer_4_ linker. The resulting scFv libraries were cloned into plasmid pCT-CON that enables display of HA/c-Myc tagged scFv antibodies on the yeast cell surface. (d) Flow cytometric analysis of antigen-specific scFv expression from organoid-derived YSD libraries constructed using the same RNA described in (a) for day 0 naïve B cells and day 4 B cells treated with either CRM_197_ carrier protein or CRM_197_-*Ft*O-PS glycoconjugate as indicated. For CRM_197_ binding (top row), yeast cells were preincubated with a nonspecific, unlabeled carrier protein, protein D (PD) from *Haemophilus influenzae*, and analyzed by flow cytometry after staining with fluorescently labeled CRM_197_ (*y*-axis) and anti-c-Myc-antibody (*x*-axis). For CRM_197_-*Ft*O-PS binding (bottom row), yeast cells were preincubated with unlabeled, aglycosylated CRM_197_, MBP, and PD carrier proteins and analyzed by flow cytometry after staining with fluorescently labeled MBP-*Ft*O-PS (*y*-axis) and anti-c-Myc-antibody (*x*-axis). All proteins were incubated at a concentration of 300 nM, and each data point corresponds to a single yeast cell. Values in each quadrant are the percentage of cells in that quadrant; red dashed-line box denotes the quadrant of interest in which immunogen binding and full-length scFv expression are both high. Results are representative of three biological replicates.

The GC is the main structure where antigen-activated B cells diversify their immunoglobulin repertoires via antibody gene mutation.^8^ Thus, to further assess the extent to which synthetic immune organoids mimic complex GC immunobiology, we investigated whether organoid-derived GC B cells manifested altered immunoglobulin gene expression in response to the different immunogens. To this end, we first evaluated clonal diversity among the different treatment groups by sequencing their expressed immunoglobulin repertoires. Specifically, RNA was isolated from GC-like organoid B cells after 4 days of *ex vivo* culture and subjected to RT-PCR to generate cDNA libraries encoding variable heavy (V_H_) and variable light (V_L_) chain domain sequences that were subsequently used to generate next-generation sequencing (NGS) libraries. Intriguingly, sequencing of the resulting NGS libraries indicated that the expressed repertoires corresponding to B cells derived from organoids exposed to either of the CRM_197_-based immunogens shared a high level of sequence convergence and were more similar to each other than to the sequenced repertoires of the initial (day 0) naïve B cell population used in preparing organoid tissues or the B cells derived from PBS-treated (day 4) organoids (Figure 6a). Nearly identical convergence results were observed with the MBP-based immunogens (Supplementary Figure S4). The observation that exposure to a common immunogen triggered the emergence of overlapping B cell repertoires that were distinctly different from control groups suggested that antigen-driven repertoire diversification was active in organoid tissues.

GC B cells are well-known to diversify their immunoglobulin variable region genes by SHM, resulting in the generation of mutant clones that have a broad range of affinities for the immunizing antigen.^37^ Therefore, we next evaluated whether the immunoglobulin gene variable regions of antigen-exposed GC organoid B cells manifested evidence of SHM. Specifically, we PCR-amplified the Sμ immunoglobulin variable locus in genomic DNA harvested from the day 0 naïve B cell population and day 4 organoid B cells and subjected the resulting amplicon libraries to NGS. Analysis of sequencing data revealed that GC organoid B cells exposed to CRM_197_-based immunogens showed a significant increase in missense mutations compared to the day 0 naïve B cell group (Figure 6b). Collectively, these results indicate that our GC organoid system was capable of reproducing select core aspects of the GC B cell phenotype, in particular diversification of immunoglobulin gene expression, consistent with previous work.^14^ While the above data demonstrate significant differences across treatments that suggest immunogen-specific SHM, at present we do not fully understand the reason behind the higher missense rate in MBP-treated organoids compared to CRM_197_-treated organoids. A possible explanation is that B cells undergo iterative rounds of somatic hypermutation and selection in GCs *in vivo*; however, in organoids, we did not examine recycling or repeated rounds of SHM. It is possible, therefore, that the final mutation rate could differ with iterative rounds and would require a more advanced immune organoid system that can induce recycling.

Given the observed diversification of immunoglobulin repertoires, we next interrogated the antigen-specificity of the B cell antibody populations. From the RNA-derived cDNA libraries, V_H_ and V_L_ genes were PCR amplified and randomly joined by a (Gly_4_Ser)_4_ linker to generate single-chain Fv (scFv) antibodies as described.^38^ Three antibody libraries were constructed: one corresponding to the day 0 naïve B cell population that was used to prepare immune organoids and one each corresponding to the day 4 B cells that were exposed to either CRM_197_ or the CRM_197_-based conjugate. Each of the resulting scFv gene libraries was cloned into the YSD plasmid pCT-CON, and the resulting plasmid libraries were used to transform *Saccharomyces cerevisiae* strain EBY100. The pCT-CON plasmid introduced a C-terminal c-Myc epitope tag to each scFv clone (Figure 6c) such that recipient yeast cells could be probed simultaneously for full-length scFv expression using an anti-c-Myc antibody and antigen-binding activity using a fluorescently labeled antigen. Accordingly, double-positive yeast cells in scatter plots were indicative of clones displaying full-length scFvs on their surface that bind the antigen of interest.

Yeast cells displaying the three different libraries were preincubated with nonlabeled protein(s) to prevent nonspecific scFvs from binding to the antigen of interest and to reduce false positive results. These cells were then interrogated for binding to fluorescently labeled versions of either the aglycosylated CRM_197_ carrier protein or the CRM_197_-*Ft*O-PS glycoconjugate by flow cytometry. Following incubation with aglycosylated CRM_197_, the library corresponding to the CRM_197_-exposed B cells was observed to contain a significant population of antigen-positive clones as evidenced by the high percentage (60.7%) of double-positive yeast cells in the library (Figure 6d). In contrast, the library corresponding to the naïve B cell population was nearly devoid of CRM_197_-binding clones with just 2.82% of the library appearing as double positive cells. A low level of CRM_197_-positive clones (21.4%) was detected in the library corresponding to the glycoconjugate-treated B cells following incubation with the same antigen, consistent with the fact that these immune organoid-derived, GC-like B cells were exposed to CRM_197_ in the context of the *Ft*O-PS antigen. Likewise, the library corresponding to CRM_197_-*Ft*O-PS glycoconjugate-treated B cells, but not day 0 naïve B cells, contained a high percentage of double-positive cells (79.4% vs 6.38%, respectively) when incubated with MBP-*Ft*O-PS glycoconjugate. Note that the mismatched MBP-based glycoconjugate was used for staining to enable detection of *Ft*O-PS-specific clones given that these organoid B cells never experienced the MBP carrier. Importantly, the library corresponding to the CRM_197_-exposed B cells, which were strongly double positive for the carrier protein alone, contained only 12.4% double positives following incubation with the MBP-*Ft*O-PS glycoconjugate, indicating a strong bias of the clones in this library for the aglycosylated CRM_197_ carrier protein. Moreover, the substantial difference in glycoconjugate binders detected in the library derived from glycoconjugate-exposed B cells compared to carrier protein-exposed B cells (79.4% vs 12.4%) suggests that the clones from the former library are specific for the *Ft*O-PS antigen.

### Discovery of High-Affinity Monoclonal Antibodies from Antigen-Exposed Organoids

**Figure 7:**
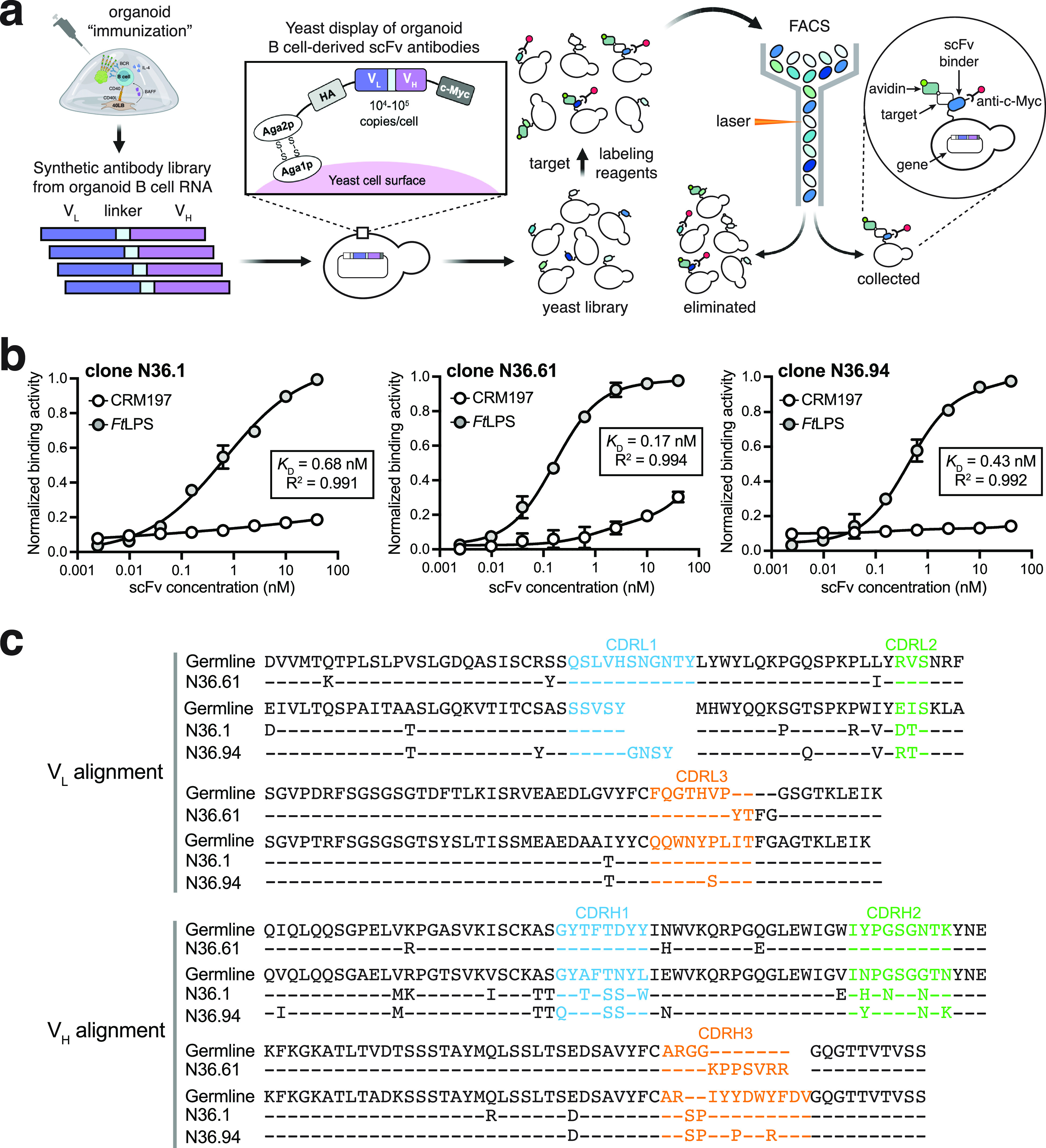
Immune organoid-enabled discovery of antigen-specific, monoclonal antibodies. (a) Schematic of YSD strategy for the isolation of antigen-specific, monoclonal antibodies from GC-like organoid B cell antibody repertoires. Libraries of V_H_ and V_L_ genes from organoid B cells were assembled as scFv antibodies in the YSD plasmid pCT-CON, after which individual yeast cells expressing antigen-specific antibody clones were isolated by FACS. (b) Antigen-binding activity and specificity for the top 3 clones, N36.1, N36.61, and N36.94, isolated from YSD library corresponding to GC-like B cells treated with CRM_197_-*Ft*O-PS glycoconjugate as determined by quantitative ELISA using *Ft*LPS (gray circles) or aglycosylated CRM_197_ (white circles) as immobilized antigen. Data are the average of three biological replicates and error bars reported as standard deviation. Inset boxes show the equilibrium dissociation constant, *K*_D_, and the coefficient of determination, *R*^2^, determined for each clone using Prism 9 software. (c) Alignment of V_L_ and V_H_ domains of anti-*Ft*O-PS antibodies along with their putative germline sequences. The putative germline amino acid structure is shown at the top with row(s) below representing mutations from the germline antibody (dash = no mutation). Complementarity determining region (CDR) 1, 2, and 3 in the variable light and heavy chains are colored blue, green, and orange, respectively, as designated by IMGT analyses.

Encouraged by these population-level observations, we proceeded to investigate whether individual antibody clones with high affinity and specificity for CRM_197_ or the *Ft*O-PS antigen could be discovered from immune organoids that had been immunized with the carrier protein or glycoconjugate, respectively (Figure 7a). To this end, we turned to the organoid-derived YSD libraries described above. These libraries, corresponding to organoids treated with either CRM_197_ or the CRM_197_ glycoconjugate, were subjected to several rounds of negative selection using magnetic-activated cell sorting (MACS) to deplete the libraries of binders to undesired targets (i.e., nonspecific proteins such as MBP or streptavidin) or to the carrier proteins in the case of libraries generated from glycoconjugate-treated organoids (see Methods for details). Then, an additional round of MACS was performed to positively select binders, with the YSD library corresponding to the CRM_197_-exposed organoids being selected against CRM_197_ and the YSD library corresponding to the CRM_197_ glycoconjugate-exposed organoids being selected against MBP bearing the *Ft*O-PS glycan. Lastly, two rounds of fluorescence-activated cell (FACS) sorting were performed with the same CRM_197_ and MBP-*Ft*O-PS antigens at a concentration of 300 nM and then 100 nM.

During the final round of FACS sorting, individual yeast cells representing putative antigen-positive clones were sorted into 96-well plates. Clonal plasmid DNA from these selected clones was used to transform the *S. cerevisiae* strain YVH10, which permits soluble expression and secretion of pCT-CON-encoded scFvs into the media supernatant.^39^ Screening of supernatants for antigen-specific binders uncovered a total of 46/96 and 62/96 clones with affinity and specificity for CRM_197_ and the *Ft*O-PS antigen, respectively, as determined by a qualitative enzyme-linked immunosorbent assay (ELISA) of YVH10 culture supernatants (Supplementary Figure S5). Three of the strongest scFv binders from each library were expressed in YVH10 cells and purified from extracellular supernatants, and then subjected to quantitative ELISA. All six clones exhibited high affinity to their respective antigen with equilibrium binding constants in the low nanomolar to subnanomolar range (Figure 7b and Supplementary Figure S6a). Importantly, the clones were also observed to be specific for their antigen with little to no cross-reactivity to either a nonspecific protein in the case of the three CRM_197_-specific scFvs or to the aglycosylated CRM_197_ carrier protein in the case of the three *Ft*O-PS-specific scFvs. Sequencing of the six clones enabled us to assign the putative germline genes and subsequently align the organoid-derived sequences with the putative germline sequences. We observed that all isolated clones exhibited 63–95% similarity with the respective germline V_H_ and V_L_ sequences (Figure 7c and Supplementary Figure S6b). Moreover, each clone contained more than 10 germline mutations, with mutations occurring both in the frameworks and complementarity determining regions (CDRs) for each. The high degree of homology and number of germline mutations observed here were on par with previous studies that have explored the immunological evolution from germline to affinity-mature antibody structures, including those against carbohydrate antigens.^40^

## Discussion

Here, using an engineered B cell immune organoid platform, we investigated the impact of designer glycoconjugate vaccine candidates on B cell maturation and signaling. In general, CRM_197_-*Ft*O-PS, a conjugate shown to elicit robust antipolysaccharide antibody titers *in vivo,* demonstrated the highest levels of BCR clustering and activation of intracellular signaling pathways in organoid B cells relative to all other immunogens tested. Downstream of these important activation signals, the CRM_197_-*Ft*O-PS-exposed B cells showed elevated levels of (i) genes important for early- and late-stage GC development including EZH2, IRF4, and BLIMP-1; (ii) cell populations generated during and post-GC formation, and (iii) immunoglobulin mutation rates reminiscent of activated B cells undergoing SHM. Importantly, the stronger polysaccharide-specific IgG response measured *in vivo* for the CRM_197_ glycoconjugate relative to the MBP glycoconjugate was recapitulated in the totality of the outputs measured from organoids. These differences could be due to CRM_197_ having more numerous immunogenic epitopes that are recognized by BCRs, thereby permitting binding by larger pools of B cells in the starting naïve populations. Indeed, the CRM_197_ carrier alone was significantly more immunoreactive in the organoid system than MBP alone, with the former stimulating BCR expression and activating BTK and pNF-κB to a much greater extent. However, these differences in immunoreactivity were not as clearly observed *in vivo*, with both carrier protein immunogens triggering comparable carrier-specific IgG titers at day 63. One reason for this discrepancy might be the fact that the mice received three doses (prime plus two boosters), whereas the organoids only received two doses of immunogen. It is also worth pointing out that the IgG responses at day 49 exhibited a trend that more closely matched the organoid data. Moreover, it should be noted that IgG titers are not a complete reflection of B cell activation *in vivo*, since an increase in pBTK and pNF-kB could still occur without a concomitant increase in IgG production. Likewise, it is possible that the cytokines released by the innate response as a consequence of both the adjuvant and the interactions with the protein carrier might enhance the B cell activation *in vivo* following immunization with MBP.^24,41^

One unexpected observation was that both the CRM_197_-*Ft*O-PS and MBP-*Ft*O-PS glycoconjugates generally triggered stronger organoid responses, especially activation of BTK and NF-κB, compared to their respective carrier proteins alone. At present, the reasons for why glycosylation boosts the strength of B cell activation by the glycoconjugates relative to the carrier protein remain unknown and are seemingly at odds with our observation that, *in vivo*, the conjugates and carriers stimulated roughly equal carrier-specific IgG responses. One possible explanation for this discrepancy is the ability of glycoconjugates to activate a pool of polysaccharide-responsive B cells in addition to the protein-responsive B cells in the initial naïve B cell populations. This population of B cells, which are responsible for the polysaccharide-specific IgG antibodies, could account for stronger B cell activation in terms of pBTK and pNF-κB without a corresponding increase in IgG titers to the carrier protein. Another possible explanation stems from the lack of T cells in our organoid system. Within organoids, T cell activation cues to B cells are present for all B cells instead of being selective for B cells displaying the appropriate antigen epitopes on their cell surface for cognate T cell recognition and binding. What this means is that the strength of B cell activation in the organoids greatly depends on the antigen’s ability to promote BCR clustering, as seen previously in the context of integrin ligand specificity.^32^ Hence, in our organoid system, where the T cell signal is binary, a protein might be at a disadvantage compared to a repetitive polysaccharide that can promote more efficient BCR clustering as we observed in our microscopy analysis of immunogen-exposed GC-like B cells (Figures 4 and 5). In contrast, the enhanced immunoreactivity of the glycoconjugate relative to the carrier was not observed *in vivo* where presumably the processing and presentation of antigen by B cells and the modulatory effect these events have on T cell help promote strong CRM_197_-specific responses regardless of whether a polysaccharide is attached or not. It is also worth noting that the measurement of IgG titers *in vivo* does not always correlate with the amount of activated B cells, since there are B cells that may react to the glycoconjugate without class-switching, making it possible to have a stronger B cell activation to the glycoconjugate in terms of pBTK and pNF-κB without a contemporaneous change in the levels of antigen-specific IgG in circulation.

The immunoglobulin repertoires from organoids were found to be responsive to treatment with the glycoconjugates, as supported by the following observations: (i) variable region mutation rates were increased, indicative of SHM; and (ii) repertoires from organoids exposed to related antigens (e.g., CRM_197_ and CRM_197_-*Ft*O-PS) were more similar than the initial naïve B cell population. Furthermore, these repertoires were shown to be enriched for antigen-specific binders, yielding immune libraries suitable for downstream antibody discovery applications. YSD was used in conjunction with these organoid-derived libraries to successfully identify sequences targeting protein (CRM_197_) and carbohydrate (*Ft*O-PS) motifs. This is especially relevant given the increasing importance in recent years of display-based approaches for antibody discovery. It has previously been shown that immune-focused libraries can be used to identify higher affinity antibodies compared to the use of naïve libraries.^42^ However, immunized libraries are inherently limited to the discovery of antibodies against the antigen or infection generating the response.^43^ Organoids have the potential to provide the benefit of an enriched, immunized library increasing the likelihood of mining clinically relevant sequences without the need to immunize living organisms. Furthermore, we anticipate the capability to sequence repertoires against large variant libraries, which could prove useful for understanding humoral response decision-making and immunodominance. How an immune response becomes biased toward one or several of the most frequent binders is an area of particular interest that we are actively investigating.

We believe that our current “T cell-free” organoid system holds great promise for learning more about B cell activation without having to account for differences in T cell activation and thus could be advantageous for antigen-specific antibody discovery, especially in the context of self-antigens. B cells that recognize a self-antigen but receive no simultaneous T cell activation signal enter a state of anergy and become nonresponsive. With CD40L T-cell signals “on”, organoids may generate stronger B cell maturation for weakly immunogenic antigens such as tumor-associated glycans. The current organoid system could also be readily used to provide a rapid, preliminary assessment of large vaccine candidate libraries. Due to the difficulty in conjugate production, including the expression, purification, and attachment of carbohydrate motifs, a small subset of vaccine candidates is normally generated to characterize the immunological response in lieu of screening large libraries of candidates that thoroughly cover every possible design configuration. However, recent advances in glycoengineering, including the use of engineered *E. coli* and their cell-free extracts for polysaccharide synthesis and conjugation,^9,10,18,44^ have made such coverage possible. A newly developed shotgun-scanning glycomutagenesis (SSGM) method leverages bacterial glycosylation to generate neoglycoprotein libraries that differ in the site of glycan attachment and spans every amino acid position.^11^ A complete SSGM library using CRM_197_ as the carrier protein for a single polysaccharide structure results in over 500 distinct conjugates, making it infeasible to systematically test all glycosylation site variants using traditional animal immunization pipelines. The number of conjugate library members can expand even further by varying other important design variables. For example, the length of the polysaccharide chain can be controllably altered by heterologous expression of different chain-length regulator genes, generating structures of varying immunogenicity.^45^ Likewise, the density of the polysaccharide epitope can be controlled by introducing additional glycan attachment sites, leading to more heavily glycosylated conjugates that elicit varied immune responses.^20^ Other advanced glycoengineering tools such as automated glycan assembly could eventually be leveraged for producing defined, pure glycans with control over composition and length.^46^ Altogether, we envision that a significant reduction in the number of animals, costs, and time can be achieved by employing organoid-based prescreening of such large conjugate libraries to identify candidates of interest for further characterization. We also imagine future organoid designs involving cocultures of B and T cells, as recently demonstrated in a top-down approach,^16^ which would open the door to *in vitro* investigations of glycoconjugate processing and presentation by B cells and the modulatory effect that these events have on T cell help.

## Materials and Methods

### Strains and Plasmids

*E. coli* strain CLM24 was used for all protein expression work. Plasmids used in this study included pGAB2 encoding the *F. tularensis* O-PS antigen biosynthesis pathway;^19^ pMAF10 encoding an hemagglutinin (HA)-tagged version of *Cj*PglB in plasmid pMLBAD;^47^ pTrc-spDsbA-MBP-GT encoding the MBP carrier protein modified at its N-terminus with the *E. coli* DsbA signal peptide and at its C-terminus with four tandem repeats of the DQNAT glycosylation motif and a polyhistidine (6xHis) tag in plasmid pTrc99A;^22^ and pTrc-spDspA-CRM_197_-GT encoding an identical construct except with CRM_197_ cloned in place of MBP. *S. cerevisiae* strain EBY100 was used for cell surface display of scFv libraries,^48^ and *S. cerevisiae* strain YVH10^39^ was used for extracellular secretion of scFv hits isolated by FACS. Plasmid pCT-CON was used for both cell-surface and extracellular expression of scFv clones^49^ and was kindly provided by Dane Wittrup.

### Glycoconjugate Expression and Purification

To prepare glycoconjugates, CLM24 cells transformed with pGAB2, pMAF10, and either pTrc-spDspA-MBP-GT or pTrc-spDspA-CRM_197_-GT were cultured in Luria–Bertani (LB) broth at 30 °C until the optical density at 600 nm (OD_600_) reached ∼0.8. At this point, *Cj*PglB expression was induced with 0.2% arabinose (w/v) for 16 h at 30 °C, and then expression of the spDspA-MBP-GT or spDspA-CRM_197_-GT carrier proteins was induced with 1 mM isopropyl β-d-1-thiogalactopyranoside (IPTG) for an additional 8 h. To prepare aglycosylated carrier proteins, a similar protocol was followed but using CLM24 cells that were transformed with only the pTrc-spDsbA-MBP-GT or pTrc-spDsbA-CRM_197_-GT plasmid and induced with 1 mM IPTG for 16 h at 30 °C. Following protein expression, cultures were spun down and stored at −20 °C. Frozen cell pellets were resuspended in 10 mL of equilibration buffer (20 mM sodium phosphate, 300 mM NaCl, 10 mM imidazole) per 50 mL culture volume. After cells were fully resuspended in solution, cells were lysed via homogenization (Avestin EmulsiFlex) through three cycles at 15000 psi. The lysate was spun down at 15000 rpm for 30 min at 4 °C. Ni-NTA resin (1 mL per 100 mL cell culture) was washed with equilibration buffer three times, added to lysate supernatant, and rotated 1 h at 4 °C. Lysate resin was applied to a 5 mL column (Pierce) followed by three column volumes (CV) of equilibration buffer and one CV of wash buffer (20 mM phosphate, 300 mM NaCl, 25 mM imidazole). Fractions were collected by addition of 1 mL elution buffer (20 mM sodium phosphate, 300 mM NaCl, 250 mM imidazole) to column. Elution fractions containing protein were pooled and buffer exchanged into phosphate-buffered saline (PBS; 137 mM NaCl, 2.7 mM KCl, 8 mM Na_2_HPO_4_, and 2 mM KH_2_PO_4_, pH 7.4) with 10K molecular weight cutoff (MWCO) protein concentrators (Pierce).

### Protein Analysis and Immunoblotting

Purified protein samples were loaded onto 10% SDS-PAGE gels (Bio-Rad) and separated at 200 V. Detection of proteins was performed by staining gels with Coomassie blue (Bio-Rad) and by immunoblot analysis. For the latter, proteins were transferred to nitrocellulose membranes (Bio-Rad), blocked with 5% (w/v) bovine serum albumin (BSA)-TBST at room temperature for at least 1 h, washed three times with TBST (TBS, 0.05% (v/v) Tween 20) for 10 min, and probed with anti-His antibody clone AD1.1.10 (Bio-Rad Cat # MCA1396GA; 1:5000 dilution), anti-*F. tularensis* LPS antibody clone FB11 (Invitrogen; Cat # MA1-21690; 1:10000 dilution), goat antimouse DyLight 800 antibody (Bio-Rad Cat # STAR117D800GA; 1:2500 dilution), and goat antirabbit StarBright Blue 700 antibody (Bio-Rad Cat # 12004161; 1:2500 dilution). Blots were detected by fluorescence using a ChemiDoc MP imager (Bio-Rad).

### Immune Organoid Fabrication

Wildtype B cells were purified from spleens harvested from 10-to-18-week-old female C57BL/6 mice (Jackson Laboratory). After red blood cell lysis, naïve B cells were isolated from splenocyte mixtures with EasySep Mouse B Cell Isolation Kit (Stem Cell Technologies). 40LB cells, which are NIH/3T3 fibroblasts genetically engineered to express CD40L and BAFF, were obtained from Dr. Daisuke Kitamura and generated as previously described.^31^ 40LB cells were cultured with high glucose Dulbecco’s Modified Eagle Medium (DMEM) medium containing 10% (v/v) FBS and 1% (w/v) penicillin streptomycin (P/S) with all components obtained from ThermoFisher Scientific. Prior to encapsulation in organoids, 40LB cells were mitotically inhibited via incubation in cell culture complete medium containing 0.01 mg/mL mitomycin C (Sigma-Aldrich) at 37 °C for 45 min. 40LB cells were then rinsed twice with 10 mL of PBS, detached with trypsin, and counted before encapsulation.

Immune organoids containing 7.5% (w/v) PEG-4MAL were fabricated using four-arm PEG-4MAL macromer with 20-kDa molecular weight with >90% purity (Laysan Bio), adhesive thiolated peptides, and dithiolated cross-linkers. PEG-4MAL macromers were initially functionalized with thiolated adhesive integrin a_4_b_1_-binding REDV peptide (GREDVGC, >90% purity; AAPPTec) with a 4:1 MAL-to peptide molar ratio for 30 min at 37 °C. Matrix metalloproteinase (MMP)-9 degradable VPM peptide (GCRDVPMSMRGGDRCG, >90% purity; AAPPTec) and nondegradable dithiothreitol (DTT) cross-linkers were combined at a 50:50 VPM-to-DTT molar ratio. All components were diluted using PBS supplemented with calcium and magnesium (PBS++, pH 7.4) with 1% (v/v) 4-(2-hydroxyethyl)-1-piperazineethanesulfonicacid (HEPES). 40000 naïve B cells and 80000 40LB stromal cells per organoid were suspended in the cross-linker solution prior to cell encapsulation. After 5 μL of PEG-4MAL macromer solution was placed in the middle of a well of a nontreated 96-well plate, 5 μL of cell-containing cross-linker solution was injected into the droplet and mixed by pipetting 5 times. Hydrogel droplets were prepared and cured for 15 min at 37 °C for complete cross-linking. Fresh Roswell Park Memorial Institute (RPMI 1640) medium supplemented with 10% (v/v) fetal bovine serum (FBS), 1% (w/v) P/S, and 10 ng/mL IL-4 (Peprotech) was then added to each immune organoid. Purified carrier proteins, with or without conjugated *Ft*O-PS antigens, in PBS were filter-sterilized using 0.2 μm syringe filters (Nalgene) and administered to organoids at a final concentration of 1.75 μM.

### Flow Cytometry Analysis

After a specified culture time, typically 4 days, cells were harvested from organoids with 1 h enzymatic degradation accomplished using a solution of 125 U/mL of collagenase type 1 (Worthington Biochemical) dissolved in serum-free RPMI medium. Gel debris was removed from cell suspensions using MultiScreen-Mesh filter plates with 96-well receiver plate (EMD Millipore). Cells were washed with PBS and then FACS buffer, which was composed of PBS++ containing 2% (v/v) FBS and 5 mM ethylenediaminetetraacetic acid (EDTA). Cells were resuspended in FACS buffer containing antibodies, incubated on ice in the dark for 45 min, and then resuspended in FACS buffer. Intracellular marker staining was performed with a one-step protocol for intracellular proteins using a Foxp3/Transcription Factor Staining Buffer Set (eBioscience). Phosphorylation signaling protein staining was performed with a two-step protocol using an Intracellular Fixation and Permeabilization Buffer Set (eBioscience). Flow cytometry data waere acquired using an Accuri C6 Flow Cytometer (BD Biosciences), FACSymphony (BD Biosciences), or LSRFortessa (BD Biosciences) and analyzed with FlowJo software. Antimouse antibodies used for organoid flow cytometry included the following: anti-CD19 clone 1D3 (BD Biosciences Cat # 565965); anti-CD19 clone 1D3 phycoerythrin-Cyanine7 (PE-Cy7) conjugate (ThermoFisher Cat # 25–0193–82); anti-CD19 clone 1D3 BUV395 conjugate (BD Biosciences Cat # 563557); anti-GL7 PE conjugate (ThermoFisher Cat # 12-5902-82); anti-GL7 Alexa Fluor 488 conjugate (ThermoFisher Cat # 53-5902-82); anti-GL7 Alexa Fluor 647 conjugate (BD Biosciences Cat # 561529); anti-EZH2 clone AC22 eFluor 660 conjugate (ThermoFisher Cat # 50-9867-82); anti-IRF4 clone 3E4 PE conjugate (ThermoFisher Cat # 12-9858-82); anti-IRF4 clone 3E4 PE-Cy7 conjugate (ThermoFisher Cat # 25-9858-82); anti-BLIMP-1 clone 5E7 (BD Biosciences Cat # 564270); anti-BLIMP-1 clone 6D3 PE-CF594 conjugate (BD Biosciences Cat # 565274); antiphospho-BTK (Tyr551, Tyr511) clone M4G3LN PE conjugate (ThermoFisher Cat # 12-9015-42); antiphospho-NF-κB p65 (Ser529) clone B33B4WP PerCP-eFluor 710 conjugate (ThermoFisher Cat # 46-9863-42); anti-CD138 clone 281–2 Brilliant Violet (BV) 711 conjugate (BioLegend Cat # 142519); anti-IgM clone eB121-15F9 FITC conjugate (ThermoFisher Cat # 11-5890-85); IgG1 clone M1-14D12 FITC conjugate (ThermoFisher Cat # 11-4015-82); LIVE/DEAD Fixable Near-IR Dead Cell Stain Kit (ThermoFisher Cat # L34976); and LIVE/DEAD Fixable Blue Dead Cell Stain Kit (ThermoFisher Cat # L23105).

### Confocal Microscopy Imaging

Immune organoids were prepared as described above. After 4 days the organoids were washed with PBS and incubated in 200 μL fixing solution (4% (v/v) paraformaldehyde in PBS) for 15 min at room temperature in the dark. Samples were then washed twice with PBS++ and permeabilized with addition of 200 μL 0.5% (v/v) Triton X-100 in PBS for 30 min. After two more washes with PBS++, the organoids were blocked with 200 μL blocking buffer (20% (v/v) goat serum in PBS++ or 20% (v/v) donkey serum in PBS++) for 30 min. For samples using a mouse IgG primary antibody, samples were blocked with Mouse-on-Mouse IgG Blocking Solution (ThermoFisher). Samples were then stainied with relevant primary antibody (1:100 dilution in 100 μL blocking buffer) overnight at 4 °C. The next day, secondary antibodies (1:100 dilution in 100 μL blocking buffer) and DAPI stain were added for 4 h on ice in the dark. Prepared immune tissues were washed twice more, stored in PBS++, and imaged using an LSM 710 confocal microscope (Zeiss) or LSM 900 confocal microscope (Zeiss). For IgM BCR quantification, four organoids of each antigen condition were imaged, with 12 single cell images per organoid, for a total of 48 cells per antigen condition. Images were analyzed in Zen (Zeiss), and IgM BCR puncta were quantified manually on single-cell images. Antimouse antiboides used for confocal imaging included the following: anti-CD19 polyclonal (ThermoFisher Cat # PA5-27442); anti-IgM II/41 clone (ThermoFisher Cat # 14-5790-82); antidiphtheria toxin polyclonal (Abcam Cat # ab151222); and anti-*Francisella tularensis* LPS FB11 clone (ThermoFisher Cat # MA1-21690). Secondary antibodies used for confocal imaging included the following: goat antirabbit IgG (H+L) Alexa Fluor Plus 488 conjugate (ThermoFisher Cat # A32731); goat antirat IgG (H+L) Alexa Fluor Plus 647 conjugate (Thermo Fisher Cat # A-21247); goat antimouse IgG (H+L) Alexa Fluor Plus 555 conjugate (Thermo Fisher Cat # A32727); donkey antirabbit IgG (H+L) Alexa Fluor Plus 488 conjugate (ThermoFisher Cat # A32790); and donkey antirat IgG (H+L) Alexa Fluor Plus 647 conjugate (ThermoFisher Cat # A48272).

### Mouse Immunization

Six-week-old BALB/c mice (Harlan Sprague–Dawley; Jackson Laboratory) were injected s.c. with 100 μL PBS (pH 7.4) alone or with purified aglycosylated or glycosylated carrier proteins. Each group was composed of three mice, and 10 μg antigen on a total protein basis was used for immunization of all groups. Protein concentration was determined by Bradford assay. Purified proteins resuspended in PBS were mixed with an equal volume of IFA (Sigma-Aldrich) before injection. After initial immunizations, boosts of identical doses were given 21 and 42 days later. Blood was obtained at study termination on day 63 via cardiac puncture. Mice were observed at 24 and 48 h after each injection for change in behavior and physical health. No abnormal responses were observed. This work was carried out under Protocol 2012-0132 approved by the Cornell University Institutional Animal Care and Use Committee (IACUC).

### Serum Antibody Titering

To determine *F. tularensis* LPS-specific antibody titers, sera from immunized mice were subjected to ELISA. Whole blood was centrifuged at 5000*g* for 10 min, and fractionated sera was stored at −20 °C. *F. tularensis* LPS (BEI resources) prepared at a concentration of 5 μg/mL in PBS was incubated overnight at 4 °C in 96-well MaxiSorp plates (Nunc Nalgene). Plates were washed three times with PBST (PBS, 0.05% (v/v) Tween-20, 0.3% (w/v) BSA) and blocked overnight at 4 °C with 5% (w/v) nonfat dry milk (Carnation) in PBS. Sera samples were serially diluted by a factor of 2 in triplicate between 1:100 and 1:12,800,000 in blocking buffer. Plates were incubated with sera for 2 h at 37 °C. Plates were washed three times and incubated for 1 h at 37 °C in the presence of one of the following HRP-conjugated antibodies: goat antimouse IgG (Abcam Cat # ab6789; 1:25000); antimouse IgG1 (Abcam Cat # ab97240; 1:25000), and antimouse IgG2a (Abcam Cat # ab97245; 1:25000). After three additional washes with PBST, 1-Step Ultra TMB (3,3′,5,5′-tetramethylbenxidine)-ELISA substrate solution (Thermo-Fisher) was added, the plate was incubated at room temperature for 30 min, the reaction was halted with 2 M H_2_SO_4_, and absorbance was quantified via microplate spectrophotometer (Molecular Devices) at a wavelength of 450 nm. Serum antibody titers were determined by measuring the lowest dilution that resulted in signal 3 standard deviations (SDs) above no serum background controls.

### Next-Generation Sequencing and SHM Analysis

After degradation of organoids on day 4 as previously described, cells were washed in PBS and processed using a RNeasy Mini Kit (Qiagen) to extract RNA. RT-PCR was performed using a SuperScript III kit (ThermoFisher) with 500 ng RNA and oligo(dT) primers according to manufacturer’s instructions. IgH and Igλ regions were PCR amplified using the resulting cDNA along with primers that anneal to the framework region of the most abundant families of Ig rearrangements, as described previously.^51^ PCR products were cleaned-up using a PCR purification kit (Qiagen) and subsequently purified from DNA gels using a gel extraction kit (QIAGEN). After DNA quantification by Qubit fluorometer (ThermoFisher), Nextera adaptors were attached by PCR reaction, and the amplicon libraries were purified 1:1 (v/v) using magnetic AMPure XP beads (Beckman Coulter). Quality control of each library was performed by the Genomics Facility of the Cornell Biotechnology Resource Center with an AATI Fragment Analyzer (Agilent). Libraries were diluted to 6 nM concentration, pooled, and sequenced using 2 × 250 bp MiSeq (Illumina). Bioinformatic analysis was performed using the analyze command of MiXCR.^52^ Paired-end sequence reads were mapped against the *Mus musculus* primary assembly GRCm38 using aligner Star 2.4.0.^53^ A pileup of the resulting sorted bam files was made in samtools for each targeted region was made filtering by quality score >20. A list of all single nucleotide polymorphisms was made using VarScan 2.3.4^54^ on each base with minimum read depth of 10 reads and tabulated per targeted region into bed-files. These bed-files were then used to create the mutation rate per kilobase for the Sμ gene by dividing the average missense per base by the average coverage of sequenced read and multiplying by 1000 to obtain the missense rate per kilobase. Primers used for immunoglobulin mutation analysis are described elsewhere.^14^

### Immunoglobulin Library Construction

Immunoglobulin V_H_ and V_L_ regions were amplified from the cDNA preparations described above and randomly paired in the scFv format via a flexible (Gly_4_Ser)_4_ linker according to previously described PCR primers and protocols.^38^ The primers were modified to insert additional nucleotides on either end of the scFv to permit homologous recombination with yeast surface display vector pCT-CON with the following designs: 5′- cgacgattgaaggtagatacccatacgacgttccagactacgctctgcag - V_L_ - (Gly_4_Ser)_4_ linker - V_H_ - ggatccgaacaaaagcttatttctgaagaggacttgtaatagctcgagat -3′. Paired V_L_/V_H_ DNA libraries were purified by the QIAquick PCR Purification Kit (Qiagen), quantified by spectrophotometer (NanoDrop), and used to transform yeast as described previously.^55^ Briefly, 4 μg of plasmid pCT-CON, which was linearized using the above overlapping nucleotide sequences, together with 12 μg of scFv library DNA was gently mixed with 400 μL electrocompetent EBY100 yeast cells, chilled on ice for at least 5 min, and electroporated at 2.5 kV and 25 μF in a 0.2 cm gap cuvette. Cells were immediately rescued in 8 mL 1:1 1 M sorbitol and 0.5 mM CaCl_2_:YPD and incubated at 250 rpm for 1 h at 30 °C. Following centrifugation at 3000*g* for 5 min, cells were resuspended in 200 mL SD-CAA media (20 g/L d-glucose, 6.7 g/L yeast nitrogen base, 5 g/L casamino acids, 5.4 g/L Na_2_HPO_4_, and 8.6 g/L NaH_2_PO_4_·H_2_O) and incubated overnight with shaking at 250 rpm at 30 °C.

### Yeast Surface Display

The surface display procedure was adapted from work described by Chen and colleagues.^56^ Here, cells were pelleted by centrifugation at 3000*g* for 5 min, resuspended in 200 mL SG-CAA media (18 g/L galactose, 2 g/L d-glucose, 6.7 g/L yeast nitrogen base, 5 g/L casamino acids, 5.4 g/L Na_2_HPO_4_, and 8.6 g/L NaH_2_PO_4_·H_2_O), and induced overnight at 250 rpm at 20 °C. The following day, cells were pelleted, washed with 1 mL PBSA (0.1% (w/v) BSA in PBS), and resuspended in 1 mL PBSA. A series of negative selections was performed by MACS to deplete the libraries of scFv clones that bound to undesired targets. Briefly, yeast cells were incubated with 4 × 10^6^ unconjugated Dynabeads Biotin Binder (ThermoFisher) under gentle rotation for 2 h at 4 °C, and nonbinding cells were collected in the flow-through after magnetic separation. This process was repeated using BSA-conjugated beads, which were prepared by incubation of 4 × 10^6^ Dynabeads and 33 pmol of purified BSA that was biotinylated using EZ-Link Sulfo-NHS-LC-Biotinylation kit (ThermoFisher) according to manufacturer’s instructions, in 100 μL PBSA for 2 h at 4 °C. Additional rounds of negative selection were performed using Dynabeads conjugated to the following biotinylated proteins: aglycosylated MBP carrier, aglycosylated *H. influenzae* PD carrier, and, for identification of *Ft*O-PS-specific binders, aglycosylated CRM_197_. Afterward, cells were pelleted, washed, resuspended in 5 mL SD-CAA, and incubated at 30 °C at 250 rpm for 16 h.

Candidate scFvs with affinity for either CRM_197_ or *Ft*O-PS were isolated by FACS. On the day of sorting, cells were incubated at room temperature for 1 h with 300 nM of biotinylated antigens, aglycosylated CRM_197_ or MBP-*Ft*O-PS conjugate, to detect positive binders and 20 nM of rabbit monoclonal anti-c-Myc (EQKLISEEDL) phospho S62 antibody (Abcam Cat # ab51156) to detect cell-surface expression of full-length scFv clones. The total volume of the reaction was calculated so that the antigen of interest was in excess by 1 order of magnitude by assuming a surface concentration of 10^5^ scFv proteins per yeast cell. After washing three times by centrifugation at 3000*g* for 3 min in 1 mL PBSA, cells were incubated with 20 nM streptavidin Alexa Fluor 488 conjugate (ThermoFisher Cat # S32354) and 20 nM of goat antirabbit Alexa Fluor 647 (Invitrogen Cat # A-21244) in the dark and under rotation for 1 h. Cells were washed four more times by centrifugation in 1 mL PBSA before sorting. FACS was performed using a FACSMelody Cell Sorter (BD Biosciences). The 1% of cells displaying the highest signals for antigen binding and scFv expression were collected, incubated in 5 mL SD-CAA media at 250 rpm at 30 °C 250 rpm, pelleted, resuspended in 5 mL SG-CAA media, and incubated overnight at 250 rpm at 20 °C. An additional round of FACS was performed as described above using 100 nM of the same biotinylated antigens, aglycosylated CRM_197_ or MBP-*Ft*O-PS conjugate, and the top 0.1% binders were isolated. For labeling controls, induced cells were incubated with (i) biotinylated antigens but in the absence of anti-c-Myc antibody; (ii) anti-c-Myc antibody and goat antirabbit Alexa Fluor 647 in the absence of biotinylated antigens, and (iii) goat antirabbit Alexa Fluor 647 only but in absence of biotinylated antigens and anti-c-Myc antibody. Sorting gates were set such that there was no overlap between the double positives and the control groups (see Supplementary Figure S7). FACS analysis was performed using FlowJo software.

### Extracellular Expression of scFv Candidates

The yeast libraries were sequentially sorted as described above until reaching a final size of ∼10^3^ cells. Plasmid DNA from each positively selected sublibrary was extracted using the Zymoprep Yeast Plasmid Miniprep II kit (Zymo Research) using ∼10^7^ cells as the initial material. To enable soluble expression and secretion of pCT-CON-encoded scFv clones into the culture supernatant, sublibrary plasmids were incorporated into *S. cerevisiae* strain YVH10^39^ by electroporation and plated in SD-CAA agar plates (1× SD-CAA, 20 g/L agar, and 182 g/L sorbitol). Single colonies were grown overnight in 5 mL of SD-CAA at 30 °C and with shaking at 250 rpm. The cells were induced by changing the media after 24 h of growth to 10 mL of SG-CAA and 0.1% (w/v) BSA, and by incubation for 48 h at 20 °C with shaking at 250 rpm. Cells were fed 1 mL of 10× nutrient stock (67 g/L yeast nitrogen base, 50 g/L casamino acids) and incubated overnight at 20 °C and with shaking at 250 rpm. Media supernatant was collected by centrifuging the cells at 3000*g* for 5 min. Individual scFv clones were purified using Pierce anti-c-Myc agarose (ThermoFisher) following manufacturer’s instructions. Following purification, scFvs were desalted and stored in PBS at −20 °C. Purity of the samples was confirmed via SDS-PAGE and Western blot analysis, while final scFv concentrations were determined using the QuantiPro BCA protein assay kit (Sigma-Aldrich).

### Analysis of scFv Candidates

Antigen was diluted to 5 μg/mL in 0.05 M NaCO_3_ buffer, pH 9.6, and refrigerated overnight at 4 °C in 96-well high-binding plates (Corning). The plates were washed three times with 200 μL PBST (PBS + 0.1% (v/v) Tween 20) per well and blocked overnight using 200 μL of 5% (w/v) nonfat dry milk in PBS per well at 4 °C. The plates were washed three more times with PBST and incubated for 2 h with 1:4 serial dilutions of 10 mg/mL purified scFvs in 100 μL of PBS per well in triplicate, with slow mixing at room temperature. The plates were washed three times to remove unbound protein and were further incubated for 1 h at room temperature with 100 μL of anti-Myc antibody conjugated to horseradish peroxidase (HRP) (Abcam Cat # ab1326) diluted 1:25,000 in PBS. The plates were washed three times with PBST followed by the addition of 100 μL per well of 1-Step Ultra TMB-ELISA substrate solution (ThermoFisher). The reaction was allowed to develop for a maximum of 30 min with incubation in the dark followed by quenching with 100 μL of 2 M H_2_SO_4_. The absorbance of each well was recorded at 450 nm in a standard plate reader (Molecular Devices).

Plasmid DNA of positive clones was extracted using the Zymoprep Yeast Plasmid Miniprep II kit (Zymo Research) and cleaned using a PCR cleanup kit (QIAgen). Plasmid DNA was used to transform electrocompetent *E. coli* strain DH5α. Single bacterial colonies were grown overnight at 37 °C and with shaking in 5 mL of LB media supplemented with 100 μg/mL of carbenicillin. Plasmid DNA from single clones was extracted using the QIAprep Spin Miniprep Kit (QIAgen, USA) and subjected to Sanger sequencing at the Genomics Facility of the Cornell Biotechnology Resource Center.

### Statistical Analysis

Statistical significance between groups was determined by one-way ANOVA with Tukey’s posthoc test or Welch’s two-sided *t*-test (*p < 0.05, **p < 0.01, ***p < 0.001, and ****p < 0.0001; ns, not significant) using GraphPad Prism 9 for MacOS software (version 9.4.1). Statistical parameters including the definitions and values of *n*, *p* values, and SDs are reported in the figures and corresponding figure legends.

## Data Availability

All data needed to evaluate the conclusions in the paper are present in the paper and/or the Supporting Information.

## Supporting Information Available

FtO-PS- and carrier protein-specific IgG titers in mice at day 49; flow cytometry gating strategy to define cell populations; flow cytometric analysis of GC-like B cell responses in synthetic organoids; sequence convergence analysis of immunoglobulin repertoire of GC-like organoid B cells; binding analysis of organoid-derived scFv antibodies; characterization and sequences of antigen-specific, monoclonal antibodies derived from immune organoids; flow cytometry gating strategy for YSD library screening (PDF)
