# Supplementary material for: Profiling Germinal Center-like B Cell Responses to Conjugate Vaccines Using Synthetic Immune Organoids
Source: ACS Cent Sci. 2023 Apr 12;9(4):787–804. doi: 10.1021/acscentsci.2c01473 (PMC10141597; doi:10.1021/acscentsci.2c01473)
Supplement: Supplementary file 1 — oc2c01473_si_001.pdf [file oc2c01473_si_001.pdf]

Supplementary Information

Profiling germinal center-like B cell responses to conjugate vaccines using synthetic immune organoids

Tyler D. Moeller[1†], Shivem B. Shah[2†], Kristine Lai[3], Natalia Lopez-Barbosa[1], Primit Desai[4], Weiyao Wang[1], Zhe Zhong[3], David Redmond[5,6], Ankur Singh[3,7*] and Matthew P. DeLisa[1,2,4,8*]

[1]Robert F. Smith School of Chemical and Biomolecular Engineering, Cornell University, Ithaca, New York 14853 USA

[2]Nancy E. and Peter C. Meinig School of Biomedical Engineering, Cornell University, Ithaca, New York 14853 USA

[3]George W. Woodruff School of Mechanical Engineering, Georgia Institute of Technology, Atlanta, Georgia 30332 USA

[4]Biochemistry, Molecular and Cell Biology, Cornell University, Ithaca, New York 14853 USA

[5]Institute for Computational Biomedicine, Weill Cornell Medicine, Cornell University, New York, NY, 10021, USA

[6]Department of Physiology and Biophysics, Weill Cornell Medicine, Cornell University, New York, NY, 10021, USA

[7]Wallace H. Coulter Department of Biomedical Engineering, Georgia Institute of Technology, Atlanta, Georgia 30332 USA

[8]Cornell Institute of Biotechnology, Cornell University, Ithaca, New York 14853 USA

[†]These authors contributed equally to this work.

*Address correspondence to: (1) Matthew P. DeLisa, Robert Frederick Smith School of Chemical and Biomolecular Engineering, Cornell University, Ithaca, NY 14853 USA. Tel: 607-254-8560; Email: md255@cornell.edu; and (2) Ankur Singh, George W. Woodruff School of Mechanical Engineering, Georgia Institute of Technology, Atlanta, Georgia 30332 USA. Tel: 404-894-5150; Email: ankur.singh@gatech.edu.

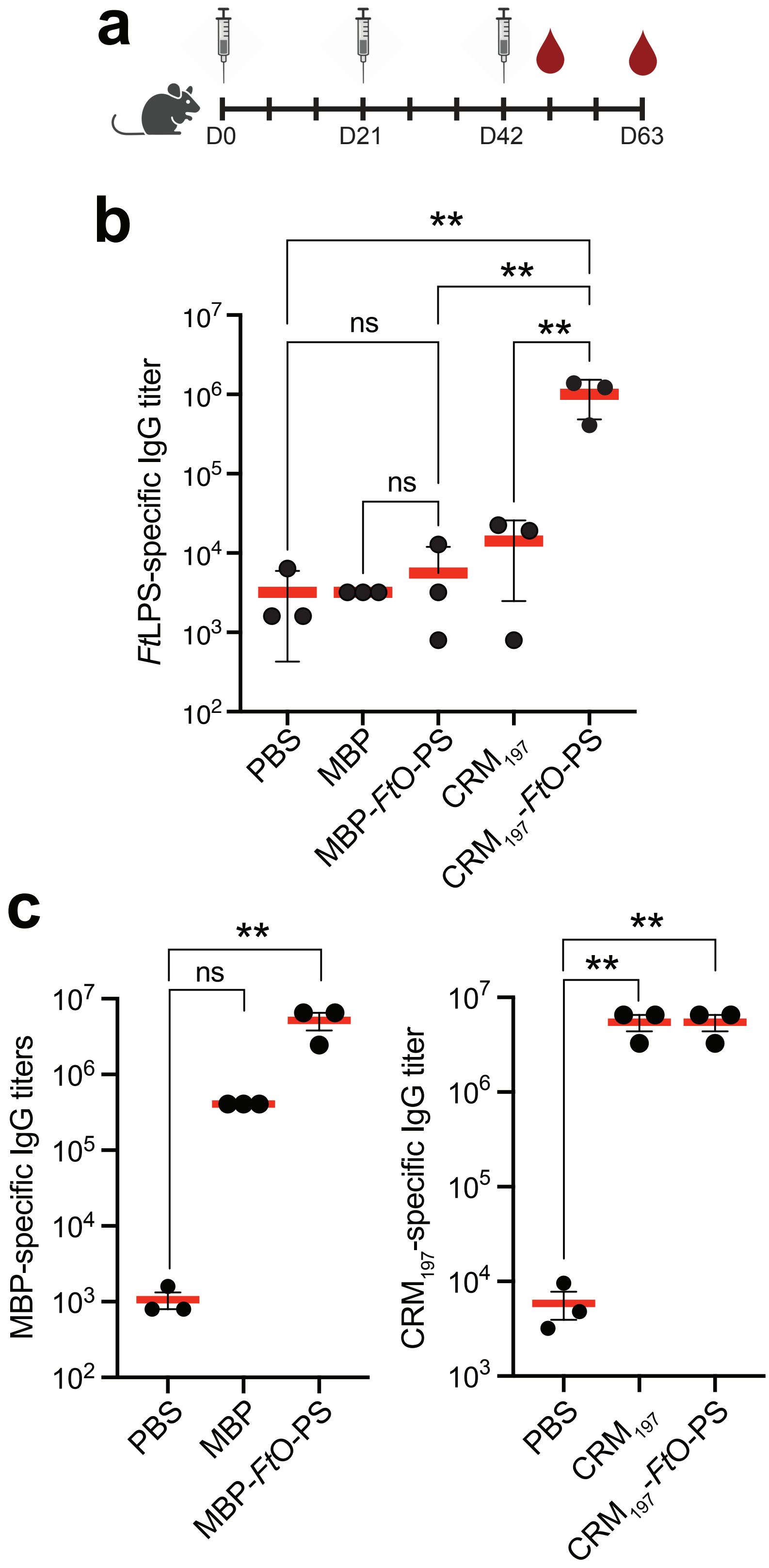

Supplementary Figure S1. Glycoconjugates differentially boost antigen-specific IgG antibody titers *in vivo*. (a) Schematic of the prime-boost immunization schedule. Mice received an initial injection on day 0 (D0) and identically formulated booster injections on days 21 and 42. Blood was drawn on days 49 and 63. (b) *Ft*LPS-specific IgG titers in day 49 serum of individual mice (black dots) and median titers of each group (red lines) measured by ELISA with *Ft*LPS as immobilized antigen. Groups of three BALB/c mice were immunized s.c. with 100 µL of PBS alone or PBS containing 10 µg of glycoconjugate (MBP-*Ft*O-PS or CRM_{197}-*Ft*O-PS) adjuvanted with IFA or 10 µg of aglycosylated carrier protein (MBP or CRM_{197}) adjuvanted with IFA. Mice were boosted on days 21 and 42 with the same doses. (c) Carrier protein-specific serum IgG titers in day 49 serum of individual mice (black dots) determined as in (a) but with MBP (left panel) and CRM_{197} (right panel) as immobilized antigens. Significant differences were determined via one-way ANOVA with Tukey's post-hoc test (*$p < 0.05$, **$p < 0.01$; ns, not significant).

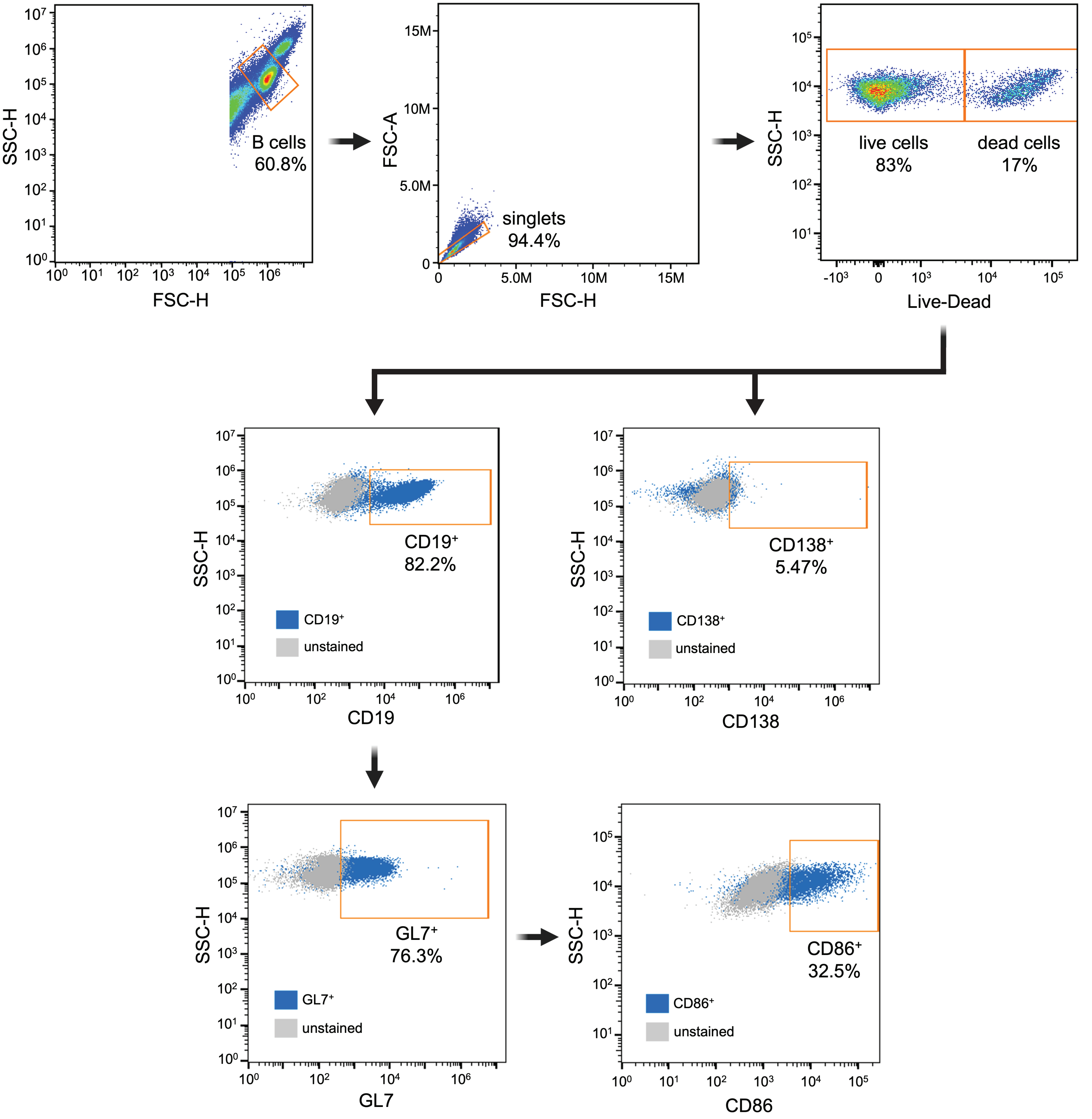

Supplementary Figure S2. Flow cytometry gating strategy to define cell populations. (a) Gating strategy is outlined beginning with size selection of lymphocytes (top left) followed by singlets (top middle) and then live cells (top right). Within the live cell population, $CD19^+$ (middle left) and $CD138^+$ (middle right) populations were defined. From the $CD19^+$ cell population, a GC-like cell population ($GL7^+$) was identified, and from the GC-like cells, LZ-like cells ($CD86^+$) were found.

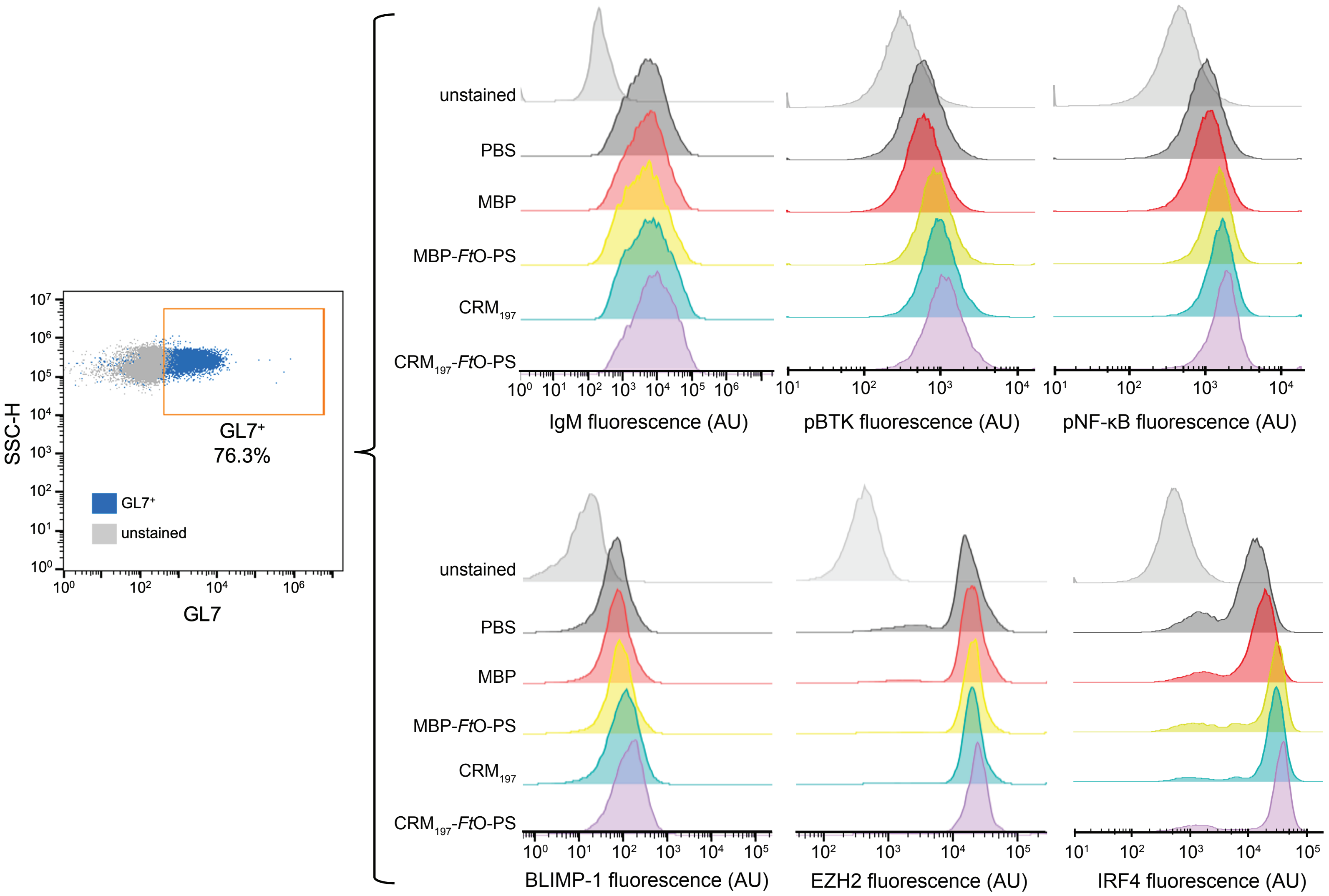

Supplementary Figure S3. Flow cytometric analysis of GC-like B cell responses in synthetic organoids. Flow cytometry histograms for expression specified markers (IgM, pBTK, pNF-κB, BLIMP-1, EZH2, and IRF4) within GL7+ cells (GC-like) with respect to PBS, MBP, MBP-*Ft*O-PS, CRM_{197}, and CRM_{197}-*Ft*O-PS. Unstained controls included in light gray.

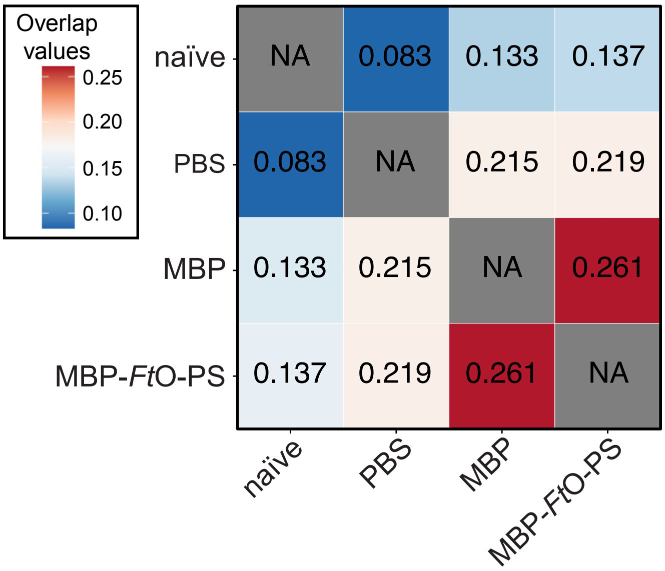

Supplementary Figure S4. GC-like organoid B cell immunoglobulin repertoire analysis. Sequence convergence of V_H repertoires as measured by Morisita's overlap index. Libraries of V_H genes were prepared from RNA of naïve B cells from day 0 prior to organoid culture (naïve) and after four days of organoid culture treated with PBS, MBP carrier protein, or MBP-*Ft*O-PS glycoconjugate.

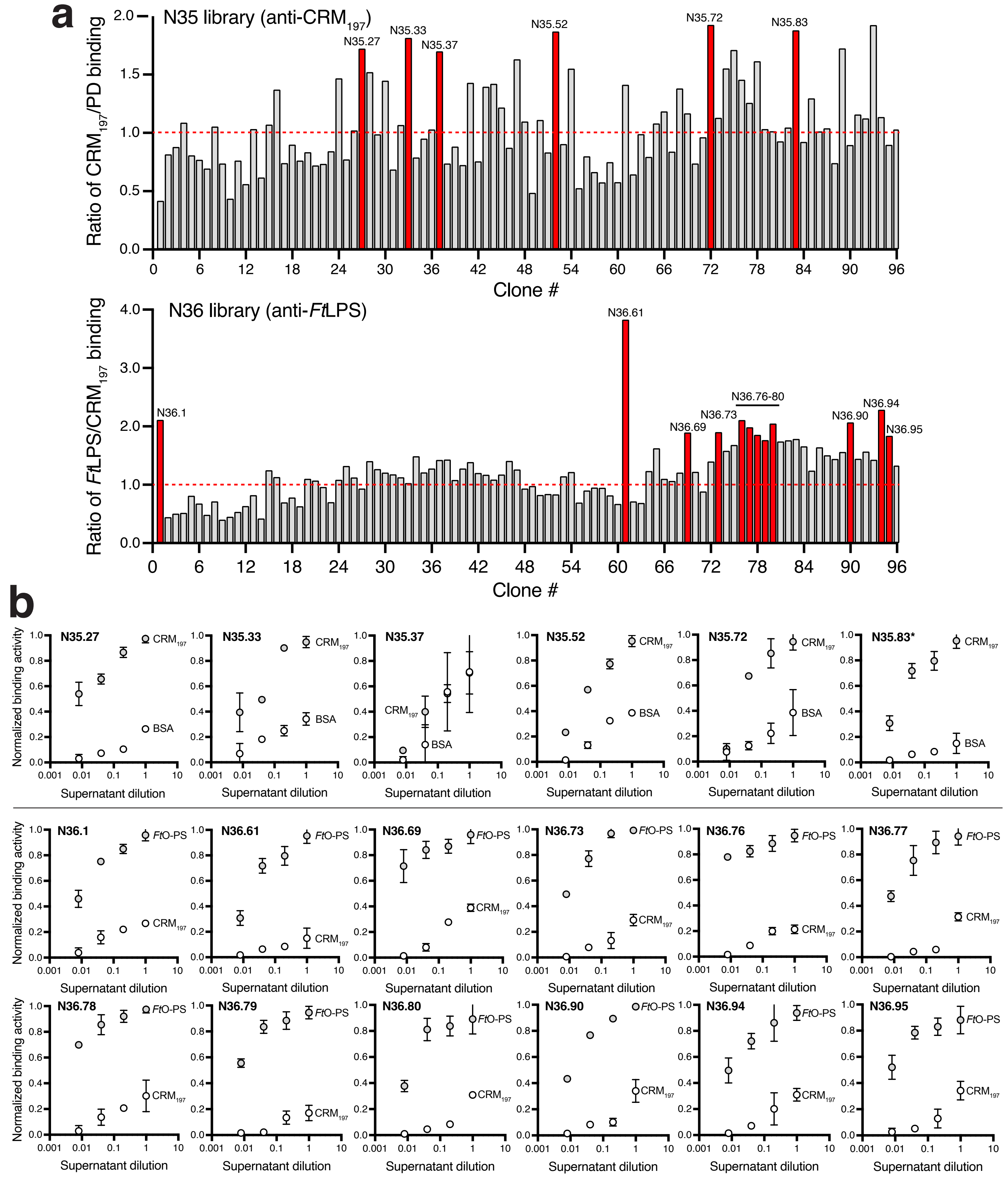

Supplementary Figure S5. Binding analysis of organoid-derived scFv antibodies. (a) Large-scale antigen-binding analysis for 96 putative binders isolated from N35 library for binding to aglycosylated CRM$_{197}$ (top panel) or N36 library for binding to CRM$_{197}$-*Ft*O-PS (bottom panel). ELISA with different immobilized antigens was performed to determine binding activity and specificity, with binding ratios determined by normalizing the binding values measured with immobilized CRM$_{197}$ or *Ft*LPS to those measured with immobilized PD or CRM$_{197}$. Ratio data are representative of three biological replicates with positive hits having ratios >1 (dashed red line). Red bars denote clones that were arbitrarily chosen for quantitative ELISA. (b) Quantitative ELISA for arbitrarily selected clones in (a). ELISA for N35 hits (top row) was performed using aglycosylated CRM$_{197}$ (gray circles) or BSA (white circles) as immobilized antigen. ELISA for N36 hits (bottom two rows) was performed with *Ft*LPS (gray circles) or aglycosylated CRM$_{197}$ (white circles) as immobilized antigen. Data are average of three biological replicates and error bars are standard deviation.

a

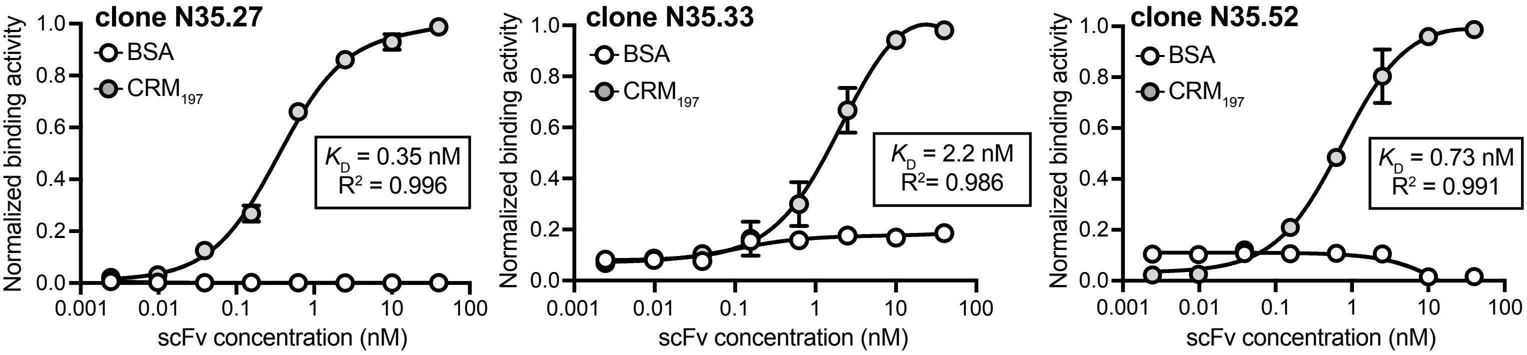

b

V_L alignment

```
                                      CDRL1
Germline  QSALTQPASVSGSPGQSITISCTGTSSDVGSYTYVSWYQQKPEQSPKLLIY
N35.27    --------------------------------NL------H-GKA---M--
N35.33    --------------------------------NL------H-GKA---M--
N35.52    ------------------------------G-NL------H-GKA---M--

          CDRL2                                  CDRL3
Germline  GASNRYTGVPDRFTGSGSATDFTLTISSVQAEDLADYHCQSYSYPSTLVFGGGT
N35.27    EG-K-PS--SN--S--K-GNTAS----GL----E---Y-C--AGS---------
N35.33    ---K-PS--SN--S--K-GNTAS----GL----E---Y-C--AGS---------
N35.52    EG-K-PS--SN--S--K-GNTAS----GL----E---Y-C--AGS---------
```

V_H alignment

```
                               CDRL1                   CDRL2
Germline  QSGAELLVPGASVKLSCKASGFNIKDYYMHWVKQRPEQGLEWIGRIDPEDGETKYAPK
N35.27    ------VK------V------YTFTG------R-A-G-----M-W-S-VS-G-N--Q-
N35.33    ------VK------V------YTFTG------R-A-G-----M-W-S-VS-G-N--Q-
N35.52    ------VK------V-------TFT-------R-A-G-----M-W---VS---N--Q-

                                                      CDRL3
Germline  FQGKATITADTSSNTAYLQLSSLTSEDTAVYYCAR------------YYYYG---VSGR
N35.27    ---RV-M-R---IS---ME--R-R-D---------APLFPIGVLAGD-----MER----
N35.33    ---RV-M-R---IS---ME--R-R-D---------APLFPTGVLAGD-----MGR----
N35.52    ---RV-M-R---IS---ME--R-R-D---------APLFPTGVLAGD---WYFDV----
```

Supplementary Figure S6. Immune organoid-enabled discovery of antigen-specific, monoclonal antibodies. (a) Antigen-binding activity and specificity for the top 3 clones, N35.27, N35.33, and N35.52, isolated from YSD library corresponding to GC-like B cells treated with aglycosylated CRM_{197} carrier protein. Quantitative ELISA was performed using aglycosylated CRM_{197} (gray circles) or BSA (white circles) as immobilized antigen. Data are the average of three biological replicates and error bars are standard deviation. Inset boxes show the equilibrium dissociation constant, K_D, and the coefficient of determination, R^2, determined for each clone using Prism 9 software. (c) Alignment of V_L and V_H domains of anti-CRM_{197} antibodies along with their putative germline sequences. The putative germline amino acid structure is shown at the top with rows below representing mutations from the germline antibody (dash = no mutation). Complementarity determining region (CDR) 1, 2 and 3 in the variable light and heavy chains are colored blue, green, and orange, respectively, as designated by IMGT analyses.

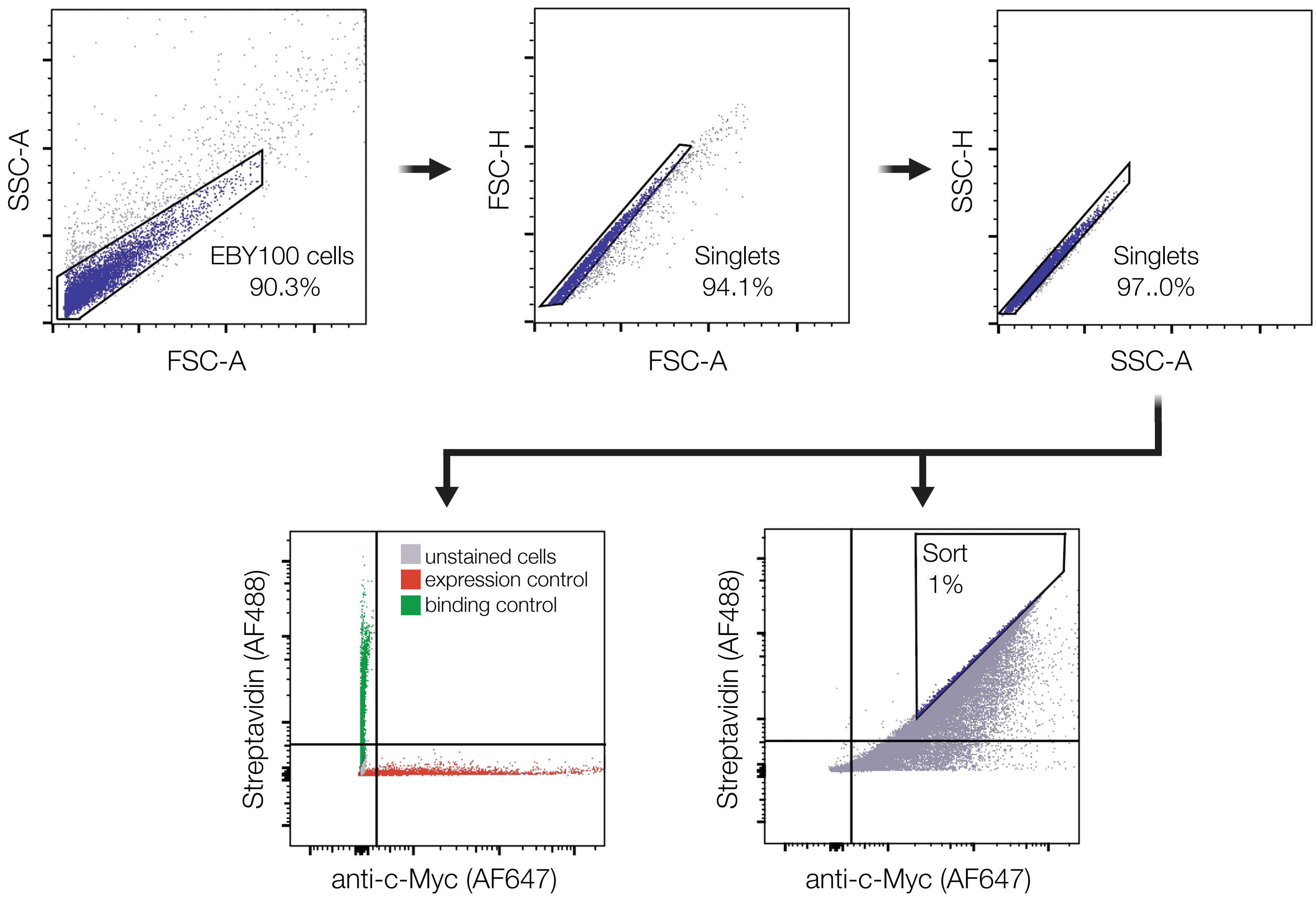

Supplementary Figure S7. Flow cytometry gating strategy for YSD library screening. Forward scatter (FSC-A) and side scatter (SSC-A) plots were used to select EBY100 yeast cells. The gated cells were sequentially narrowed to single cells using FSC-A/FSC-H and SSC-A/SSC-H plots. Cells incubated with streptavidin Alexa Fluor 488 (AF488) and anti-rabbit Alexa Fluor 647 (AF647) were used to determine the unstained population (grey). Similarly, cells labeled with anti-c-Myc AF647 (red) or streptavidin AF488 (green) were used to determine the single-labeled populations. The sorting gate was set along the diagonal for expression normalization and without any overlap with the single-labeled or unstained populations.
